# Exosomes displaying native EGF enhance doxorubicin’s therapeutic efficacy and reduce cardiotoxicity

**DOI:** 10.1186/s12951-025-04002-9

**Published:** 2026-01-27

**Authors:** Liang Mao, Longtao Qian, Xin Zhou, Keran Niu, Zelin Li, Shuyun Wang, Jiankang Xie, Wen Zhou, Xitong Dang

**Affiliations:** 1 The Key Laboratory of Medical Electrophysiology of Ministry of Education, Medical Electrophysiological Key Laboratory of Sichuan Province, Collaborative Innovation Center for Prevention and Treatment of Cardiovascular Disease of Sichuan Province, Institute of Cardiovascular Research, Department of Cardiovascular Medicine, The First Affiliated Hospital of Southwest Medical University, Southwest Medical University, Luzhou, 646000 Sichuan China; 2 Institute of Cardiovascular Research, Southwest Medical University, 1-1 Xianglin Road, Longmatan District, Luzhou, 646000 Sichuan China

**Keywords:** Doxorubicin, EGF ligand, EGFR, Exosomes, Targeted delivery, Cardiotoxicity

## Abstract

**Background:**

Doxorubicin (DOX) is one of the most potent chemotherapeutic agents for cancer treatment. However, its cumulative and often irreversible, life-threatening cardiotoxicity significantly limits its clinical applications. While strategies like dose reduction, iron chelation, and liposome encapsulation have aided in mitigating cardiotoxicity to certain extent, they are associated with decreased therapeutic efficacy and potential cancer relapse, the risk of developing secondary malignancy, and the incidence of the Hand-foot syndrome. Exosomes (Exo) are naturally occurring nanoparticles that can be engineered to display targeting moieties on their surface, thereby enhancing drug delivery efficacy. We aimed to develop an exosomal DOX formulation targeting broad epidermal growth factor receptor (EGFR) variants to enhance its anti-tumor efficacy and minimize cardiotoxicity.

**Results:**

The native 53-amino-acid EGF was decorated on the surface of exosomes by genetically engineering exosome-producing A549 cells. The EGF-Exo was effectively internalized by tumor cell lines in a manner dependent on EGFR expression levels, and exhibited enhanced accumulation in xenograft A549 tumors relative to the heart, with minimal cardiac accumulation. When loaded with DOX, these engineered exosomes were rapidly internalized, inducing higher apoptosis in A549 cells compared to liposomal-DOX. Upon systemic administration in an A549 xenograft mouse model, EGF-Exo-DOX exhibited enhanced accumulation in tumors relative to the heart, with minimal cardiac accumulation, significantly reducing tumor burden, mitigating DOX-induced cardiotoxicity, and exhibiting no tumorigenic effects. This favorable therapeutic profile is primarily attributed to DOX-induced apoptosis.

**Conclusions:**

Our findings demonstrate that tumor-derived exosomes engineered with EGF on their surface enable targeted drug delivery to tumors with high EGFR expression. Although the exosomes modestly increase cell proliferation in vitro, the EGF-Exo-DOX formulation exhibits enhanced tumor accumulation relative to the heart, minimal cardiac uptake, and shows no tumorigenic effects in vivo. Compared to Lipo-DOX, a widely used clinical formulation of liposomal DOX in China, EGF-Exo-DOX demonstrates superior cellular uptake, greater induction of tumor cell apoptosis, and improved anti-tumor efficacy. These results highlight the potential of engineered exosomes as a targeted drug delivery platform for patients with EGFR-overexpressing tumors.

**Graphical abstract:**

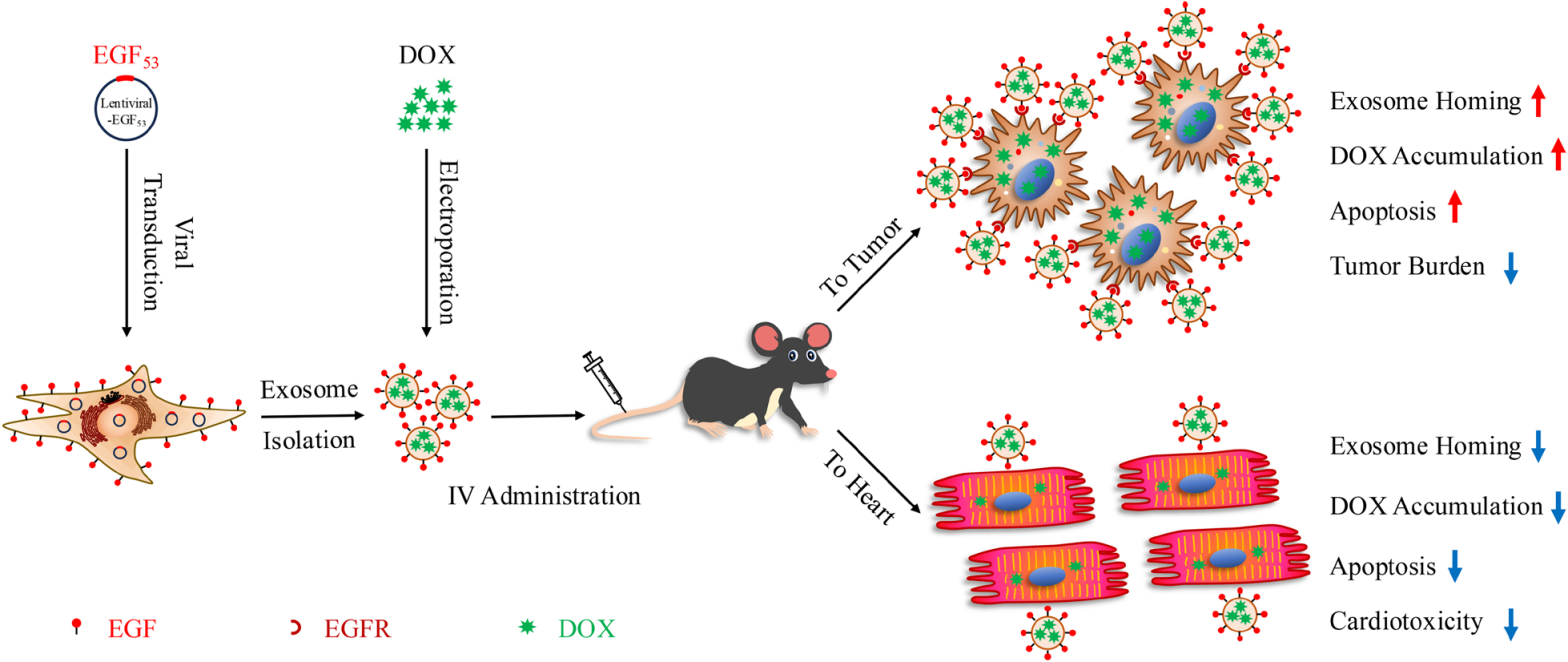

**Supplementary Information:**

The online version contains supplementary material available at 10.1186/s12951-025-04002-9.

## Introduction

Doxorubicin (DOX), a member of anthracycline family, is the most effective chemotherapeutic agent used to treat sarcomas of soft tissue and bone, hematological cancer, and solid tumors of many organs [1]. It has significantly increased the survival of cancer patients. Despite its high anti-tumor potency, DOX has a dose-dependent cardiotoxicity ranging from asymptomatic subtle structural and functional changes of the heart to irreversible cardiomyopathy, leading to congestive heart failure and even death [2, 3]. The mechanisms underlying DOX’s cardiotoxicity result from an interplay among many factors, including inflammation, oxidative stress, mitochondrial damage, energetic stress, intracellular Ca^2+^ overload, iron-free radical production, DNA damage, and myocyte membrane injuries, among others [4–6]. Regrettably, only a few drugs targeting these signaling pathways are currently in clinical use, and their clinical efficacy requires additional validation.

Liposomal encapsulation of DOX, as seen in clinically used formulations such as Doxil and Myocet, is a widely adopted strategy to enhance therapeutic efficacy and reduce cardiotoxicity. These liposomal formulations prolong DOX’s half-life, improve tumor-targeted delivery, and minimize systemic and cardiac toxicity [7–9]. The therapeutic efficacy can be further enhanced by surface conjugation of ligands/antibodies targeting receptors like EGFR [10–12]. Despite improved drug delivery efficiency and overall safety profile, the therapeutic efficacy of liposomal DOX remains only marginally superior to free DOX [10, 11]. Additionally, the risk of hand-foot syndrome (HFS) and other side effects associated with liposome limits its widespread clinical use [13–15].

Exosomes are nanosized, lipid bilayer enclosed structure that transport various signaling molecules, including proteins, nucleic acids, and lipids, to recipient cells, facilitating intercellular communication [16]. Importantly, due to exosomes’ excellent biocompatibility, ability to traverse biological barriers, low immunogenicity, large cargo loading capacity, and ease of surface functionalization for active targeting, they have been widely studied as drug delivery vehicles for various therapeutic reagents [17–21]. DOX has been loaded into exosomes either directly via co-incubation (simple, with saponin, or followed by freeze-thaw cycles), electroporation, or indirectly by incubating it with exosome-producing parental cells [19, 22, 23]. The resulting exosomal DOX demonstrated rapid cellular uptake and enhanced anti-tumor efficacy in cell lines, while showing no cardiac accumulation upon systemic delivery, in contrast to free DOX and liposomal DOX [9, 24–26]. Naturally occurring exosomes possess intrinsic ability to target specific cells or tissues [27, 28]; however, the precise molecular mechanisms driving this natural tropism remain incompletely understood. As a result, leveraging the natural tropism for targeted drug delivery remains significantly constrained. Exosome membrane proteins can be modified to display targeting moieties either directly through chemical coupling or indirectly by engineering exosome-producing parental cells. Tumor-penetrating peptides (e.g., iRGD), homing peptides, and aptamers have been successfully incorporated onto exosome surfaces, which enhances the delivery of DOX to tumors while reducing its accumulation in the heart, leading to increased efficacy of tumor suppression and reduced cardiotoxicity [9, 24, 29, 30].

The upregulation of EGFR expression is linked to increased proliferation, metastasis, chemotherapy resistance, and poor prognosis [31–33]. However, it offers an ideal target for precision drug delivery. The GE11 homing peptide, which binds to EGFR with moderate affinity, has been displayed on the surface of exosomes, enabling targeted delivery and enhancing anti-tumor efficacy of the encapsulated drugs [34–36]. Despite this enhanced therapeutic efficacy and low mitogenic effect, GE11, as a synthetic peptide, faces several challenges, including potential immunogenicity, a short half-life, weak to moderate binding specificity and affinity, and less efficient internalization, which restrict its broad clinical applicability [37–39]. To this end, a novel anti-tumor therapeutic strategy was evaluated in a tumor cell xenograft mouse model, combining the inherent tumor-targeting ability and antigen-specific immune response of tumor cell-derived exosomes, surface display of EGF to broadly target EGFR variants, and encapsulation of DOX within the exosomes. The engineered exosomes were taken up by EGFR-expressing cell lines in vitro in an EGFR-level dependent manner, and exhibited enhanced accumulation in xenograft A549 tumors relative to the heart, showing minimal cardiac accumulation. Importantly, when loaded with DOX, EGF-Exo were more efficiently internalized by A549 cells and induced a higher rate of apoptosis than Lipo-DOX, a chemotherapeutic agent commonly used in clinical practice in China. In vivo, systemic administration of EGF-Exo-DOX in A549 xenograft mice led to a significant reduction in tumor burden and exhibited lower cardiotoxicity compared to Lipo-DOX. Additionally, no evidence of tumor metastasis or neoangiogenesis was observed in mice treated with EGF-Exo-DOX. This favorable therapeutic profile primarily driven by DOX’s apoptosis-inducing activity, suggests EGF-Exo-DOX formulation as a promising off-the-shelf therapy for EGFR-positive tumors.

## Materials and methods

**Ethics** All animal studies were performed in accordance with the Chinese National Guidelines for the Use and Care of Experimental Animals. All protocols for animal studies were reviewed and approved by the Ethics Committee of Southwest Medical University (XNYD20220820-002).

**Reagents** Lentiviruses encoding LAMP2b and EGF-LAMP2b were constructed by FUBIO Inc. (Suzhou, China). Vybrant DiD cell-labeling solution (V-22887), CellROX Oxidative Stress Reagent (C10443), Dead Cell Apoptosis Kit with Annexin V APC and SYTOX Green (V35113), and RevertAid First Strand cDNA Synthesis Kit (K1622) were purchased from ThermoFisher (US). TUNEL Assay Kit was purchased from Beyotime (C1090, Shanghai, China). Doxorubicin (E2516) and Thiazolyl Blue (S6821) were products of Selleck Chemicals Inc. (US). Doxorubicin Hydrochloride Liposome Injection (Lipo-Dox), a clinically approved liposomal formulation of DOX in China, was obtained from CSPC Pharmaceutical Group Limited (Shijiazhuang, China). Masson Stain Kit was purchased from Nanjing Jiancheng Biotech Inc. (Nanjing, China). Primers were synthesized by Qingke Biotech Inc. (Chengdu, China). Isoflurane was the product of RWD life science Inc. (Shenzhen, China). NU/NU Nude Mice were from Charles River Inc. (Beijing, China). All other chemicals, unless stated otherwise, were from Sigma-Aldrich. All cell lines including A549, HEK293, MCF-7, H9C2, HS578T, A375, and HeLa were purchased from Cell Bank of Shanghai Institute of Biochemistry and Cell Biology, CHINA.

**Plasmid construction** To target exosomes to EGFR, the coding sequence of EGF was fused in-frame between AA35 and 36 of the human LAMP-2b gene by overlapping polymerase chain reaction (PCR), as previously described [18]. The PCR products EGF-LAMP2b or LAMP2b alone was cloned into the pLVX-IRES-ZsGreen1 plasmid. The identity of plasmids was confirmed by DNA sequencing (Qingke, Chengdu, China). Lentiviruses encoding EGF-LAMP2b and LAMP2b were packaged and purified by FUBIO Inc. (Suzhou, China).

**Exosomes isolation and Dox loading** A549 cells were transduced with LAMP2b and EGF-LAMP2b lentiviruses, respectively. The stable cell lines overexpressing LAMP2b (control (CON)-A549) and EGF-LAMP2b (EGF-A549) were established by sorting the green fluorescent protein (GFP)-positive cells. CON-A549 and EGF-A549 cell lines were cultured in Dulbecco’s Modified Eagle Medium (DMEM) containing 10% Fetal Bovine Serum (FBS) and 1% Penicillin-Streptomycin (Pen/Strep). When the cell density reached 80%, the medium was refreshed with serum-free DMEM. Cells were continued to culture for an additional 48 h, and the conditioned medium (CdM) was collected for exosome isolation. Briefly, the CdM was centrifuged at 2000 × g for 10 min at 4 °C to remove dead cells, followed by centrifugation at 10,000 × g for 30 min at 4 °C to eliminate cell debris. The supernatant was then ultracentrifuged at 100,000 × g for 70 min at 4 °C. The pellet was washed with PBS and subjected to another ultracentrifugation at 100,000 × g for 70 min at 4 °C. The resulting pellet was resuspended with 1 mL of PBS and stored at −80 °C for downstream applications.

To encapsulate DOX into exosomes, CON-Exo or EGF-Exo (1 × 10^8^ particles) and DOX (3.30 µg) were mixed in 1 mL of PBS containing 50mM Trehalose [31]. The mixture was then electroporated in 0.4-mm cuvettes, with 200µL per cuvette, applying 100 V for 5 ms, repeated twice, followed by 20 V for 50 ms, repeated five times. Subsequently, the electroporated mixture was ultracentrifuged at 100,000× g for 70 min at 4 °C. The pellet, containing exosomes loaded with DOX, was resuspended in 100µL of PBS and stored at −80 °C for future use.

The amount of DOX loaded in EGF-Exo-DOX was measured using high-performance liquid chromatography (HPLC). Standard (Std) curves were generated to establish the relationship between absorbance and DOX concentration for accurate quantification. This curve was created by plotting absorbance values against a concentration range of 0 to 25 µg DOX dissolved in 1 mL of methanol. A Diamonsil Plus C18 column (50 × 4.6 mm, 5 μm) was utilized, with a mobile phase consisting of ultrapure water containing 2% glacial acetic acid and methanol in a 1:1 (v/v) ratio. The flow rate was set at 1 mL/min. Peaks were monitored using fluorescence detection at an excitation wavelength of 480 nm and an emission wavelength of 550 nm. The concentration DOX loaded into Exo was calculated using the formula: [Exo-DOX] = [Std-DOX]× (Peak area of Exo-DOX)/(Peak area of Std-DOX).

## A549 xenograft tumor mouse model

The A549 tumor cell xenograft mouse model used was established using a previously described method [19]. In brief, A549 cells were cultured in DMEM containing 10% FBS and 1% Pen/Strep. When the cell density reached 90%, the cells were trypsinized and adjusted to a density of 1 × 10^7^ cells/mL. Subsequently, 100µL of the cell suspension was subcutaneously injected into the right groin of Nu/Nu mice. Two weeks after the inoculation, mice received tail vein injection for a total of 10 doses with one of the following treatment: CON-Exo or EGF-Exo (2 × 10^8^ particles in 200µL), CON-Exo-DOX or EGF-Exo-DOX (2 × 10^8^ particles in 200µL, containing 1.40 µg DOX), lipo-DOX (containing 1.40 µg DOX in 200µL), 1.40 µg free DOX, or NS. Tumor size was measured immediately before exosome injection and daily thereafter using a Vernier caliper. Tumor volume was calculated using the modified ellipsoidal formula: Volume = 0.5× (Length× Width^2^).

## IVIS in mice tumor

To investigate the distribution of exosomes after intravenous injection, CON-Exo and EGF-Exo were labeled with Vybrant CM-DiD and then administered to mice via the tail vein. The distribution of fluorescent exosomes was detected using the In Vivo Imaging System (IVIS). CON-Exo or EGF-Exo (2 × 10^8^ particles) and 4µL of CM-DiD cell-labeling solution (1mM) were mixed in 1 mL of PBS, followed by incubation for 15 min at 37 °C. Subsequently, the mixture was ultracentrifuged at 100,000× g for 70 min at 4 °C to remove free CM-DiD probes. The resulting pellet was suspended in 100µL of PBS. The DiD-labeled CON-Exo and EGF-Exo were then diluted 100-fold with PBS. The MFI was measured using a fluorescence microplate reader (excitation: 644 nm; emission: 665 nm). Subsequently, DiD-labeled CON-Exo and EGF-Exo (2 × 10^8^ particles) were individually administered to the A549 xenograft tumor mice via the tail vein, two weeks after the A549 cell inoculation. Twenty-four hours after the exosome injection, the mice were anesthetized with isoflurane, and the gross fluorescent profile was detected by IVIS. Subsequently, the mice were euthanized and systemically perfused with PBS from the left ventricle. The organs, including the tumor, heart, lung, kidney, liver, and spleen, were harvested, and their fluorescence was detected using IVIS.

## Echocardiography and histological analysis

After ten doses of exosomes administration, the mice were anesthetized with isoflurane and cardiac function were assessed by echocardiography using Vevo 3100 (Visual Sonics, Canada) equipped with a 40-MHz MS400D probe and a high-frequency ultrasound system. Left Ventricular Ejection (LVEF), Left Ventricular Fractional Shortening (LVFS), Left Ventricular Mass (LV Mass), and Heart Rate were analyzed using Vevo LAB software. After the echocardiography, the mice were euthanized and systemically perfused with PBS from the left ventricle. Tumors, hearts, lungs, livers, spleen, and kidneys were then harvested, rinsed in PBS, weighed, and embedded in an optimal cutting temperature (OCT) medium. Frozen sections of 6.0 μm were fixed in 4% paraformaldehyde and stained with H&E (Hematoxylin-eosin), Masson’s trichrome, CD31 (a marker for endothelial cells), and Matrix Metalloproteinase-9 (MMP-9). All images were evaluated using ImageJ software.

**Apoptosis analysis** A549 or H9C2 cells were seeded in a 6-well plate (1 × 10^6^ cells/well) and cultured in complete DMEM containing 10% FBS for overnight. The medium was replaced with DMEM containing either CON-Exo-DOX or EGF-Exo-DOX (2 × 10^7^ particles) or free Doxorubicin. After 4–8 h, the cells were trypsinized, stained for apoptotic cells with the Dead Cell Apoptosis Kit using Annexin V APC and SYTOX Green, and analyzed using flow cytometry. To compare the effects of EGF-Exo-DOX and Lipo-DOX on cellular uptake and apoptosis, A549 cells were seeded as described above. After overnight incubation, cells were treated with EGF-Exo-DOX, CON-Exo-DOX or Lipo-DOX at equivalent DOX doses for 20, 40, and 90 min, followed by confocal immunofluorescence imaging to analyze cellular internalization. At 4 h post-treatment, cells were stained using a TUNEL kit according to the manufacturer’s protocol. Fluorescence images were acquired and analyzed using ImageJ software.

## Statistical analysis

The data were analyzed using GraphPad Prism software (GraphPad Software, San Diego, CA). For the comparison of multiple groups, one-way ANOVA was conducted, followed by a post hoc Tukey’s test. In contrast, comparisons between two groups were executed using Student’s t-test. The results are presented as ‘Mean ± SD’ derived from a minimum of three independent experiments in duplicates, with * *P* < 0.05, ** *P* < 0.01, *** *P* < 0.001, and **** *P* < 0.0001.

## Results

### Characterization of exosomes displaying EGF

Increased EGFR expression is associated with poor prognosis in many epithelial-origin tumors, yet it offers a valuable target for drug delivery [32]. To target exosomes to cells bearing EGFR, the coding sequence of the EGF was fused in-frame between AA35 and 36 of human LAMP2b gene. The fusion gene was cloned into pLVX-IRES-ZsGreen1, a bicistronic lenti-viral plasmid. Lentiviruses carrying the EGF-LAMP2b or LAMP2b control (CON) were used to transduce lung carcinoma epithelial cells, A549 (Figure S1A), from which exosomes (EGF-Exo and CON-Exo) were prepared by ultracentrifugation. Both EGF- and CON-Exo exhibited a spherical shape by transmission electron microscopy (TEM) (Figure S1B), with an average diameter of 123 nm for CON-Exo and 125 nm for EGF-Exo by Nanoparticle Tracking Analysis (NTA) (Figure S1B). Both exosomes were positive for exosome markers, Alix, TSG101, and CD9 by Western blot. Importantly, an immunoreactive EGF band could be detected in EGF-Exo, but not in CON-Exo (Figure S1C). Moreover, the average diameter of EGF-Exo remained stable for up to 21 days when stored at −80 °C (Figure S1D). These results suggest that the EGF can be displayed on the surface of exosomes, which share similar physical characteristics as the non-targeted native exosomes.

### EGF-Exo targets EGFR-expressing cells in an EGFR dose-dependent manner

**Fig. 1:**
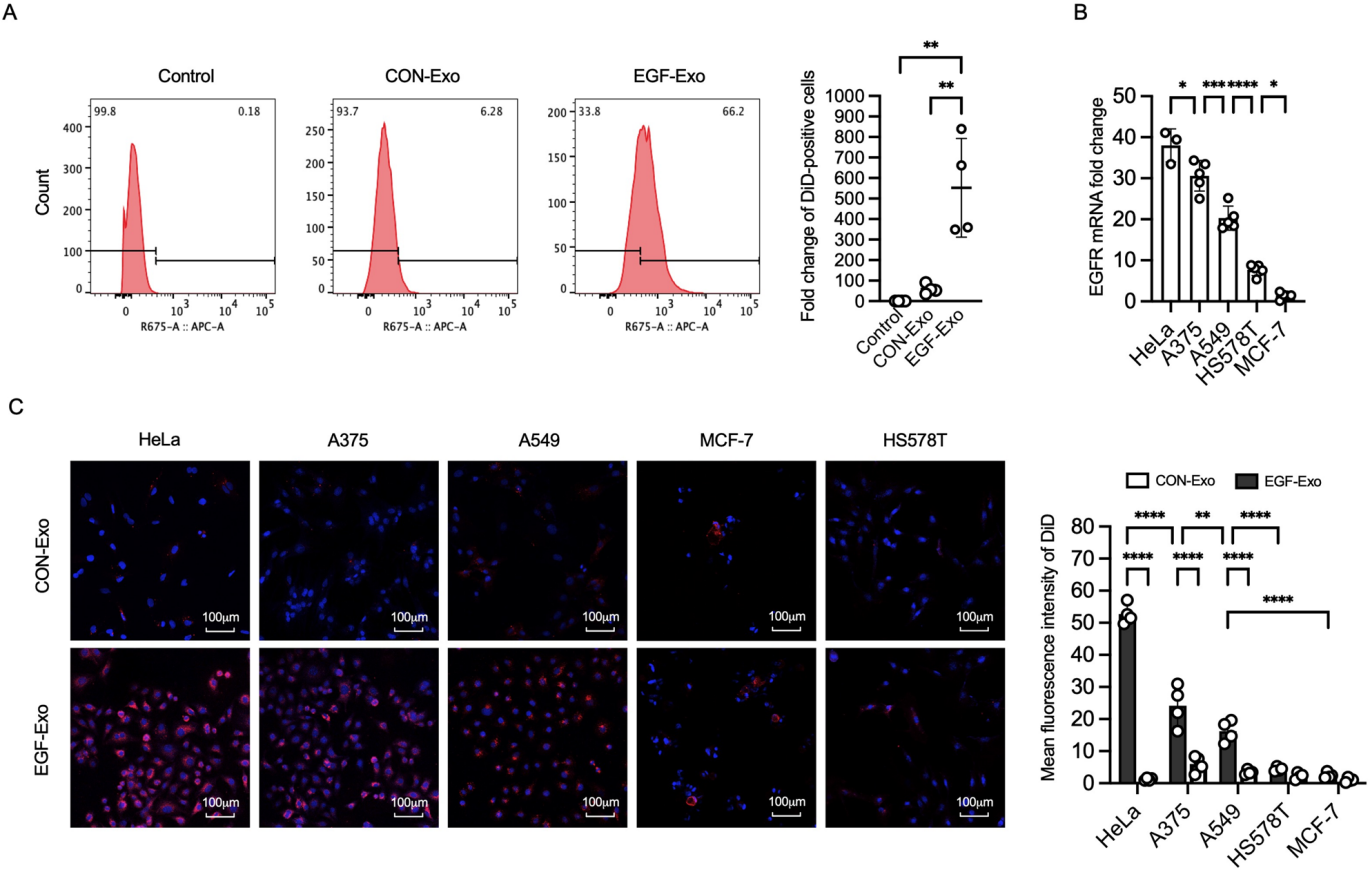
EGF-Exo targets EGFR-expressing cells in an EGFR level-dependent manner. Exosomes labelled with DiD-Dye were incubated with HeLa, A375, A549, HS578T and MCF-7 cells for 8 h, respectively. (**A**) Representative flow cytometry histogram and quantitative analysis illustrating the distribution of fluorescence in A549 cells. (**B**) The levels of EGFR expression in different tumor cells by qPCR (*n* = 5). (**C**) Representative fluorescence images and quantitative analysis of cells after treatment with CON-Exo or EGF-Exo. DiD-labelled exosome (red), and DAPI-stained nuclei (blue). Scale = 100 μm. Gene expression was normalized to that of GAPDH. Experiments were repeated three times in duplicates. Data are expressed as “Mean ± SD”, with *p < 0.05, **p < 0.01, ***p < 0.001, and ****p < 0.0001

To evaluate the targeting capability, exosomes-derived A549 cells were labeled with Vybrant DiD Cell-Labeling dye (DiD). The labeled exosomes were diluted 100-fold in PBS, and their MFI was measured using a fluorescence microplate reader (excitation: 644 nm; emission: 655 nm). The results showed similar MFI between CON-Exo and EGF-Exo, indicating equivalent labeling efficiency (Figure S1E). Exosomes were then incubated with A549 cells for 8 h, and exosome internalization was analyzed by flow cytometry. As shown in Fig. 1A, the percentage of DiD-positive cells was significantly higher in cells incubated with EGF-Exo (66.2%) compared to CON-Exo (6.28%) at 8 h. To assess whether EGF-Exo uptake correlates with EGFR expression levels, EGF-Exo and CON-Exo derived from HEK293 cells were incubated with HeLa, A375, A549, HS578T, and MCF-7 cells, which express progressively lower levels of EGFR (Fig. 1B) [33, 34], at a ratio of 8.3 exosomes per cell for 8 h. Exosome internalization was analyzed using confocal microscopy. As shown in Fig. 1C, the mean fluorescence intensity (MFI) in the EGF-Exo group was 42-fold higher in HeLa cells, 4-fold higher in A375 cells, and 4.7-fold higher in A549 cells compared to the CON-Exo group. In contrast, no significant difference in MFI was observed between the EGF-Exo and CON-Exo in cells with lower EGFR expression (HS578T, MCF-7)(Fig. 1B and C). These results indicate EGF-Exo, regardless of their source cells, specifically targets EGFR-expressing cells in an EGFR-level-dependent manner.

### In vivo biodistribution of EGF-Exo

**Fig. 2:**
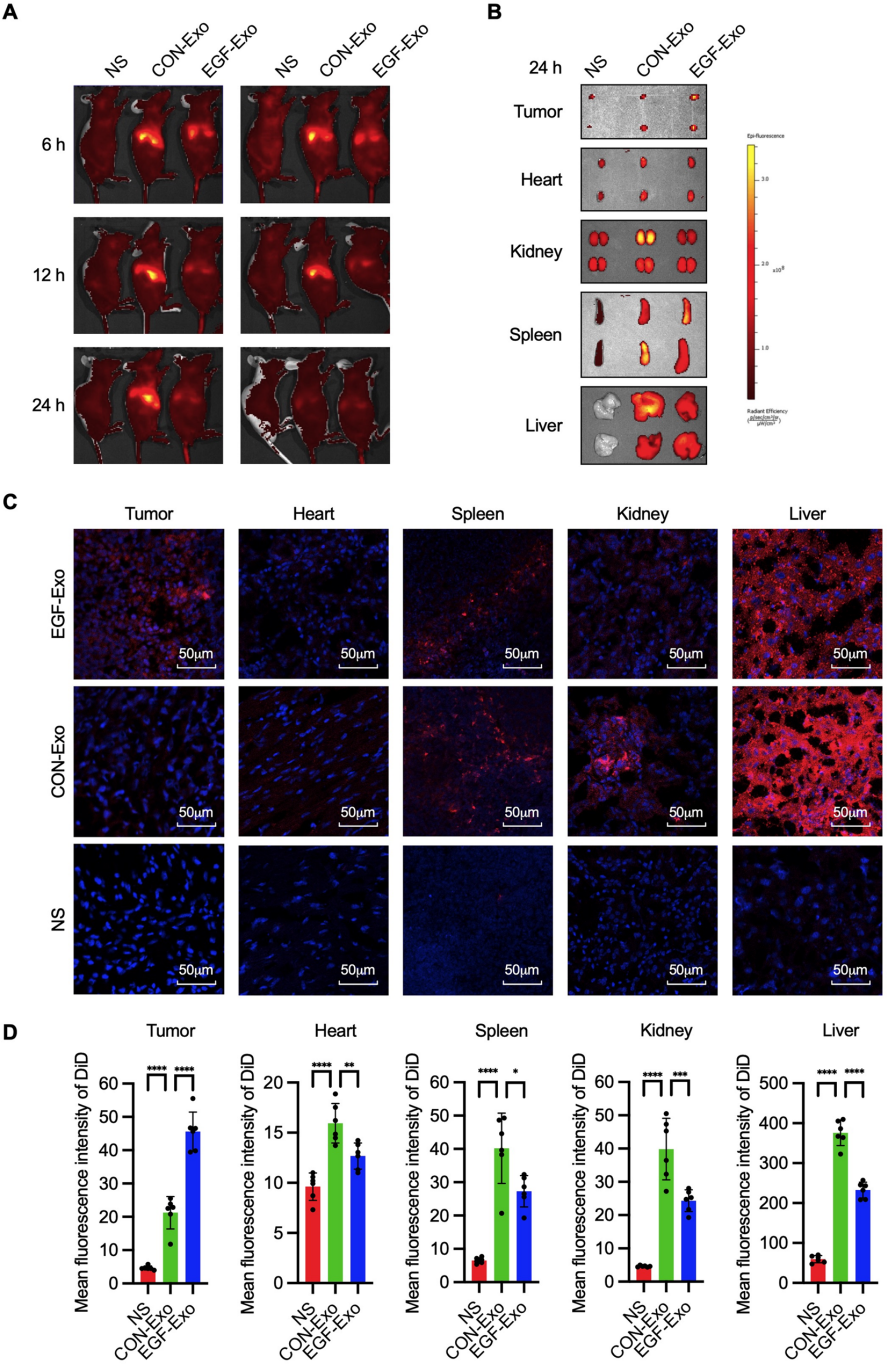
Distribution of EGF-Exo in A549 tumor cell xenograft mice. A549 tumor cell xenograft mice were established. Once the tumor volume reached approximately 60 mm³, mice were given tail-vein injections of 100µL NS or PBS containing 2 × 10⁸ DiD-labelled CON-Exo or EGF-Exo. (**A**) Whole-body fluorescence images at 6, 12, and 24 h by IVIS system. **(B**) Fluorescence images of tumors, hearts, spleens, kidneys, and livers at 24 h (*n* = 2 mice per group). (**C**) Representative fluorescence images of the cross-sections of tumors, hearts, spleens, kidneys, and livers by confocal microscopy (*n* = 6, with 3 distinct microscopic fields were selected and analyzed per mouse). (**D**) Quantitation of the mean fluorescent intensity in (**C**). DiD-labeled exosomes (red), and DAPI-stained nuclei (blue). Scale bar = 50 μm. Experiments were repeated three times in duplicates. Data are expressed as " Mean ± SD “, with * *P* < 0.05, ** *P* < 0.01, ***p < 0.001, and ****p < 0.0001

We have shown that EGF-Exo targets tumor cells in an EGFR level-dependent manner. We next investigated the in vivo tumor targeting and biodistribution of exosomes in an A549 cell xenograft mouse model using an in vivo imaging system (IVIS). One hundred (100) µL of normal saline (NS) or an equal volume of PBS containing 2 × 10^8^ DiD-labeled CON-Exo or EGF-Exo was administered to each mouse through tail-vein injection, and their biodistribution was analyzed at 6, 12, and 24 h post-injection. The results demonstrated that, although the overall fluorescent intensity was high in the liver area for mice receiving both EGF-Exo and CON-Exo, it was significantly lower in mice receiving EGF-Exo compared to CON-Exo in the whole-body IVIS (*n* = 2) (Fig. 2A). Importantly, ex vivo IVIS revealed that, compared to mice receiving CON-Exo, the MFI was significantly higher in tumors but lower in the liver, kidney, and spleen in mice receiving EGF-Exo. The MFI in the hearts was comparable between the two groups (*n* = 2)(Fig. 2B). This tissue and organ distribution pattern was validated in tissue cross-sections using confocal microscopy. As shown in Fig. 2C and D, the MFI in tumors increased progressively in the order of normal saline (NS), CON-Exo, and EGF-Exo. Conversely, the MFI in the heart, spleen, kidney, and liver increased in the order of NS, EGF-Exo, and CON-Exo (*n* = 6, with three distinct microscopic fields analyzed per mouse). Taken together, these results demonstrate that exosomes displaying the EGF show enhanced targeting of EGFR-positive tumors.

### EGF-Exo increased cell viability in vitro but did not promote tumor growth in vivo

To assess the potential tumorigenic risk of EGF-Exo, A549 cells were incubated with CON-Exo or EGF-Exo (1 × 10^8^ particles/mL) for 24 h. Cell viability and cell migration were evaluated by MTT and cell scratch assay, respectively. EGF-Exo significantly increased cell viability by 26.69% (Figure S2B), indicating enhanced cell proliferation in vitro, but showed no effect on cell migration (Figure S2A), compared to PBS- or CON-Exo-treated cells. To investigate whether this cell proliferative effect translated in vivo, CON-Exo or EGF-Exo (2 × 10^8^ particles in 200µL) was administered to A549 xenograft-bearing mice via tail vein injection every other day for a total of 10 doses. Immunofluorescence analysis of xenograft tumors revealed comparable levels of human CD31 (a marker of angiogenesis) and MMP9 (a marker associated with tumor invasion and metastasis) across all treatment groups (Figure S2C, S2D) [35, 36]. These findings suggest that while EGF-Exo promotes cell proliferation in vitro, no tumorigenic effects were observed in vivo, as it did not enhance tumor growth, angiogenesis, or invasive potential.

### EGF-Exo-DOX enhances anti-tumor potency by inducing apoptosis

To evaluate the anti-tumor efficacy of DOX delivered via EGFR-targeted exosomes, DOX was encapsulated into EGF-Exo and CON-Exo by electroporation to generate EGF-Exo-DOX and CON-Exo-DOX. Briefly, exosomes (1 × 10^8^) were mixed with DOX (3.30 µg) in 1mL PBS containing 50mM Trehalose, electroporated in 200µL aliquots, purified via ultracentrifugation to remove non-encapsulated DOX, and resuspended in 100µL sterile PBS. The concentration of encapsulated DOX (Exo-DOX) was determined using high-performance liquid chromatography (HPLC) in conjunction with a DOX standard curve (Std-DOX) at a wavelength of 480 nm [37]. The concentration of Exo-DOX was calculated using the formula: concentration of Exo-DOX = (concentration of Std-DOX) × (peak area of Exo-DOX)/(peak area of Std-DOX). Based on the formula, the DOX loading efficiency was 21.21% (0.70 µg/3.30 µg), equivalent to 1.29nmol free DOX (i.e., 1 × 10^8^ EGF-Exo-DOX in 100 µL) (Figure S3A, S3B). Additionally, the morphology and average diameters before (EGF-Exo, left panel) and after DOX loading (EGF-Exo-DOX, right panel) showed no significant changes (Figure S3C) as analyzed by TEM and NTA, indicating the integrity of EGF-Exo-DOX.

**Fig. 3:**
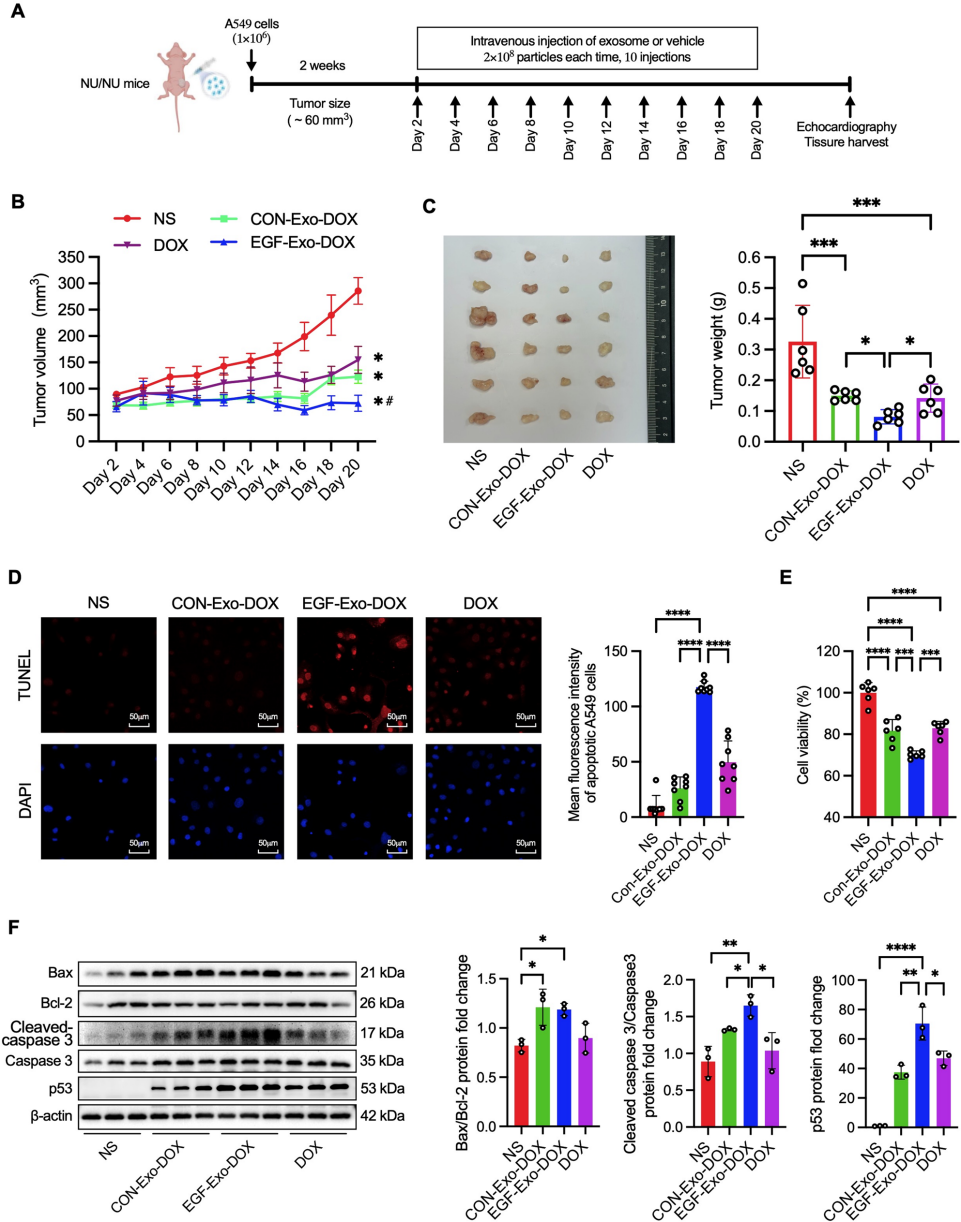
EGF-Exo-DOX exhibits enhanced anti-tumor potency. Six-week-old nude mice were subcutaneously inoculated with A549 cells (1 × 10⁶) in the groin area. Approximately 2 weeks later, mice were treated via tail vein injection every other day for a total of 10 doses with CON-Exo-DOX or EGF-Exo-DOX (each contains 2 × 10⁸ exosomes, equivalent to 1.40 µg DOX), or 1.40 µg of free DOX. (**A**) Schematic presentation of the therapeutic regimen. (**B**) Dynamic changes of tumor volume during the treatment (*n* = 6). (**C**) Gross images and quantitation of tumor weights after the treatment (*n* = 6). (**D**) Representative fluorescence images and MFI showing TUNEL-positive A549 cells treated for 8 h with either NS, CON-Exo-DOX, EGF-Exo-DOX, or free DOX. TUNEL-positive cells (red), DAPI-stained nuclei (blue), and the scale bar = 50 μm. (**E**) Cell viability of A549 cells after the above treatments by MTT (*n* = 6). (**F**) The expression of apoptotic proteins Bax, Bcl-2, p53, cleaved Caspase3, Caspase3, and β-Actin by Western blot. The expression of all target proteins was normalized to that of β-Actin. All experiments were repeated 3 times in duplicates. Data were expressed as " Mean ± SD “, with * *P* < 0.05, ** *P* < 0.01, *** *P* < 0.001, and **** *P* < 0.0001

To assess the impact of EGF-Exo-DOX on cell apoptosis, A549 cells (3 × 10⁵ cells/well) were seeded in a 6-well plate and incubated for 8 hours with 200µL of either normal saline (NS), CON-Exo-DOX, EGF-Exo-DOX (each containing 2 × 10⁸ particles, equivalent to 1.40 µg DOX, or 1.29µM), or 1.29µM free DOX for 8 hours. Apoptosis was evaluated using the TUNEL assay. Compared to cells treated with NS, the MFI was significantly higher in cells treated with CON-Exo-DOX, EGF-Exo-DOX, and free DOX, with EGF-Exo-DOX exhibiting the highest intensity (Fig. 3D). Consistently, compared to cells treated with NS, cell viability was significantly decreased in cells treated with CON-Exo-DOX, EGF-Exo-DOX, and free DOX, with the lowest levels observed in the EGF-Exo-DOX group (Fig. 3E). To investigate the mechanisms driving increased apoptosis, A549 tumor cell xenograft mice were injected via the tail vein every other day for a total of 10 doses with one of the following treatments: CON-Exo-DOX or EGF-Exo-DOX (2 × 10^8^ particles in 200µL, containing 1.4 µg of DOX), free DOX (1.4 µg in 200µL), or normal saline (NS, 200µL) (Fig. 3A), and apoptosis-related proteins in xenograft tumors were analyzed by Western blot. p53 expression levels were significantly higher in mice treated with CON-Exo-DOX, EGF-Exo-DOX, or free DOX, compared to NS-treated mice, with the highest levels observed in the EGF-Exo-DOX group. A similar trend was observed for the ratio of cleaved Caspase-3 to total Caspase-3. Additionally, the Bax/Bcl-2 ratio was significantly higher in mice treated with CON-Exo-DOX or EGF-Exo-DOX, but not free DOX, compared to NS-treated mice (Fig. 3F). These findings indicate that DOX exerts its therapeutic effect by inducing apoptosis, and the enhanced antitumor potency of EGF-Exo-DOX is attributed to its increased cellular uptake.

### EGF-Exo-DOX inhibits tumor growth with increased potency and shows no tumorigenic effects

To assess the therapeutic efficacy of EGF-Exo-DOX on tumors, A549 tumor cell xenograft mice were treated via tail vein injection every other day for a total of 10 doses with one of the following treatments: CON-Exo-DOX or EGF-Exo-DOX (2 × 10^8^ particles in 200µL, containing 1.4 µg DOX), free DOX(1.4 µg in 200µL), or NS (200µL)(Fig. 3A). The overall well-being, body weight, and tumor volume had been monitored throughout the treatment. After assessment of cardiac function by echocardiography as shown below, mice were then sacrificed, and tumors were excised and weighed. The results indicated that, while the overall well-being and body weight were comparable across all five groups, tumor volumes and tumor weights were smallest in mice treated with EGF-Exo-DOX, followed by CON-Exo-DOX, DOX, and NS (Fig. 3B and C).

To evaluate the potential tumorigenic risk of EGF-Exo-DOX, the expression of human CD31 and MMP-9 was assessed by immunofluorescence in xenograft tumors, lungs, spleens, livers, kidneys, and hearts of mice. The results showed that CD31 and MMP-9 expression levels in tumors were similar across all treatment groups (Figure S4). However, MMP-9 expression was significantly elevated in the lungs, spleens, and livers of mice treated with free DOX compared to those treated with NS, CON-Exo-DOX, or EGF-Exo-DOX, while levels were comparable in the hearts and kidneys across all groups (Figure S5). These findings suggest that EGF-Exo-DOX does not enhance the expression of tumorigenic markers in normal tissues and may reduce the off-target pro-metastatic effects seen with free DOX.

### EGF-Exo-DOX exhibits reduced cardiac DOX accumulation and preserved cardiac function

**Fig. 4:**
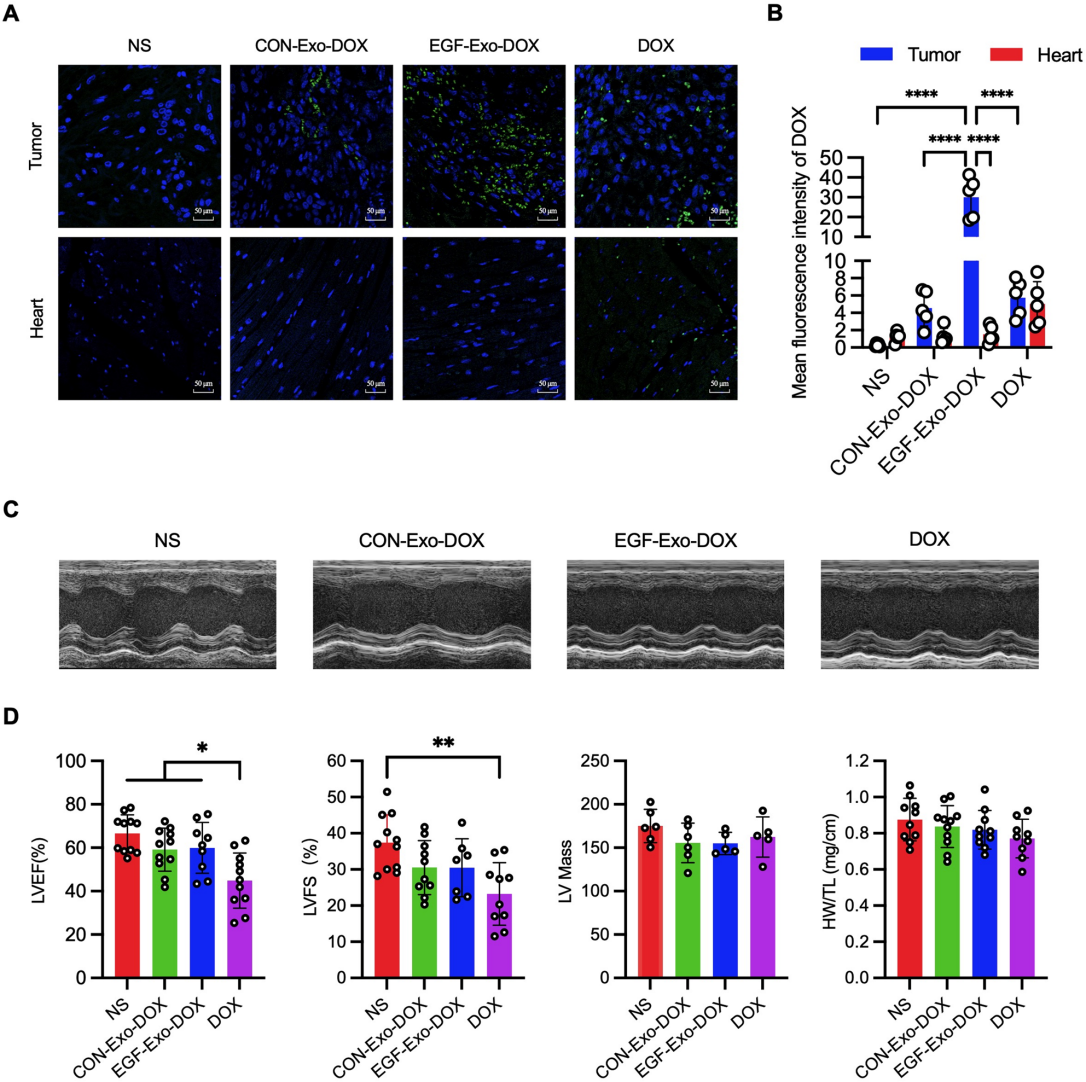
EGF-Exo-DOX delivers DOX to tumors more efficiently than to the heart and mitigates DOX-induced myocardial systolic dysfunction. (**A**) Representative confocal fluorescence images showing DOX uptake (green) in tumors and hearts of xenograft mice after treatment with NS, EGF-Exo-Dox, CON-Exo-DOX, or free DOX. DOX (green) and DAPI-counterstained nuclei (blue). Scale bar = 50 μm. (**B**) Quantification of the MFI of DOX in cross-sections of tumor and heart tissues. (**C**) Representative echocardiograms of tumor-bearing mice treated with NS, EGF-Exo-DOX, CON-Exo-DOX, or DOX (*n* = 11). (**D**) Quantitation of LVEF, LVFS, the ratio of heart weight/tibia length (mg/cm), and LV Mass in (**C**). Data are expressed as “Mean ± SD”, with *p < 0.05, **p < 0.01, ****p < 0.0001

It is well known that the cumulative and dose-dependent cardiotoxicity of DOX hampers its clinical applications. To evaluate DOX accumulation in the heart, heart cross-sections were examined using confocal microscopy at a wavelength of 480 nm, with DOX appearing green, as illustrated in Fig. 4A and B. The MFI of DOX in tumors decreased progressively in the order of EGF-Exo-DOX, CON-Exo-DOX, free DOX, and NS. In the heart, the MFI of DOX was comparable between EGF-Exo-DOX and CON-Exo-DOX groups, and both were significantly lower than that in the free DOX group. Additionally, the MFI of DOX in the hearts of EGF-Exo-DOX-treated mice was significantly lower than in their tumors (Fig. 4A and B). These findings indicate that EGF-Exo-DOX shows enhanced delivery of DOX to tumors relative to the heart.

To assess the cardiotoxicity of EGF-Exo-DOX, cardiac function was evaluated by echocardiography in A549 cell xenograft mice at the end of the 10th administration of EGF-Exo-DOX. The results showed that, compared to NS treatment, mice treated with free DOX exhibited a 35% reduction in LVEF, while those receiving EGF-Exo-DOX or CON-Exo-DOX displayed a comparable LVEF, with only a 14% reduction. Similarly, LVFS decreased by 38% in mice receiving free DOX compared to NS treatment, whereas it remained comparable between mice receiving EGF-Exo-DOX and CON-Exo-DOX, with only an 18% reduction. In contrast, no significant differences were observed in the ratio of heart weight to tibia length among all 4 groups (Fig. 4C and D).

### EGF-Exo-DOX mitigates DOX-induced cardiotoxicity through multiple mechanisms

**Fig. 5:**
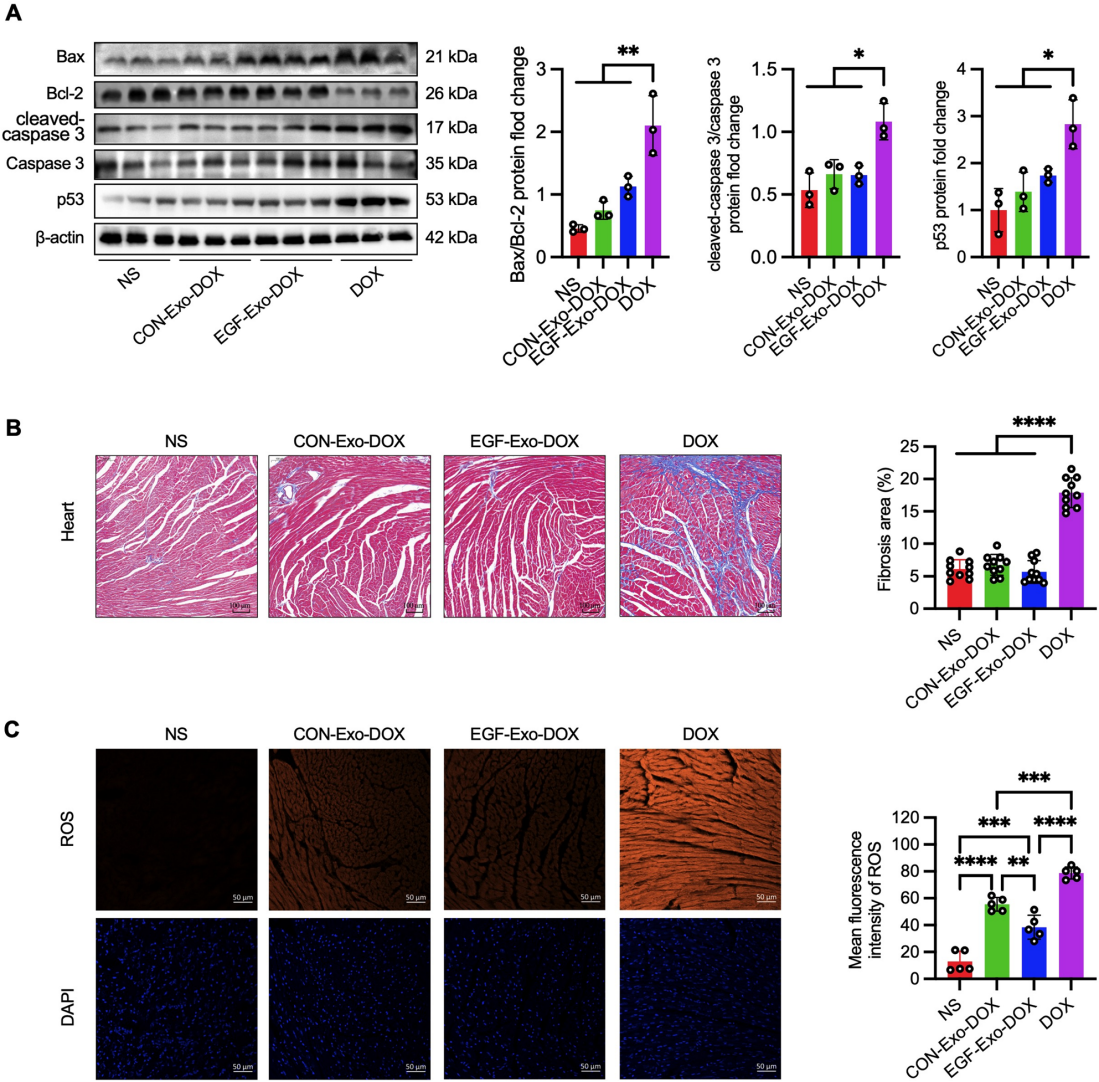
EGF-Exo-DOX ameliorates DOX-induced myocardial injury and fibrosis. (**A**) The expression of apoptosis-related protein Bax, Bcl-2, cleaved Casepase3, Caspase3, p53, and β-Actin by Western blot. (**B**) Representative images and quantitative analysis of Masson staining of the cross-sections of heart tissue, scale bar = 100 μm. (**C**) Representative fluorescence images and quantitation of ROS levels in frozen sections of heart tissue (*n* = 5). Scale bar = 50 μm. The expression of all target protein was normalized to that of β-Actin. All experiments were repeated 3 times in duplicates. Data are expressed as " Mean ± SD “, with * *P* < 0.05, ** *P* < 0.01, *** *P* < 0.001, and **** *P* < 0.0001

Apoptosis, oxidative stress, and free radical generation are among the predominant mechanisms involved in DOX-induced cardiotoxicity [35]. To investigate the potential mechanisms underlying the decreased cardiotoxicity of exosomes targeting EGFR, the expression of apoptosis-associated proteins, cardiac fibrosis, and reactive oxygen species (ROS) production were assessed in the left ventricles of A549 tumor xenograft mice treated with CON-Exo-DOX, EGF-Exo-DOX, free DOX, or NS. The results showed that Bax/Bcl-2 ratio increased progressively across treatments with NS, CON-Exo-DOX, EGF-Exo-DOX, and free DOX, although differences among the first three groups were not statistically significant. Additionally, the ratio of cleaved Caspase-3 to total Caspase-3 and p53 expression levels were significantly higher in mice treated with free DOX compared to those treated with NS, CON-Exo-DOX, or EGF-Exo-DOX (Fig. 5A). The degree of cardiac fibrosis in mice treated with CON-Exo-DOX or EGF-Exo-DOX remained at baseline levels, similar to that observed with NS treatment, and was significantly reduced compared to mice receiving free DOX (Fig. 5B). Additionally, ROS generation was significantly reduced in mice treated with CON-Exo-DOX compared to free DOX treatment, which further decreased in those treated with EGF-Exo-DOX (Fig. 5C). Collectively, our results indicate that EGF-Exo-DOX reduces DOX accumulation in the heart, thereby decreasing DOX-induced cardiomyocyte apoptosis, oxidative stress, and myocardial fibrosis.

### EGF-Exo-DOX mitigates DOX-induced cardiomyocyte injury

**Fig. 6:**
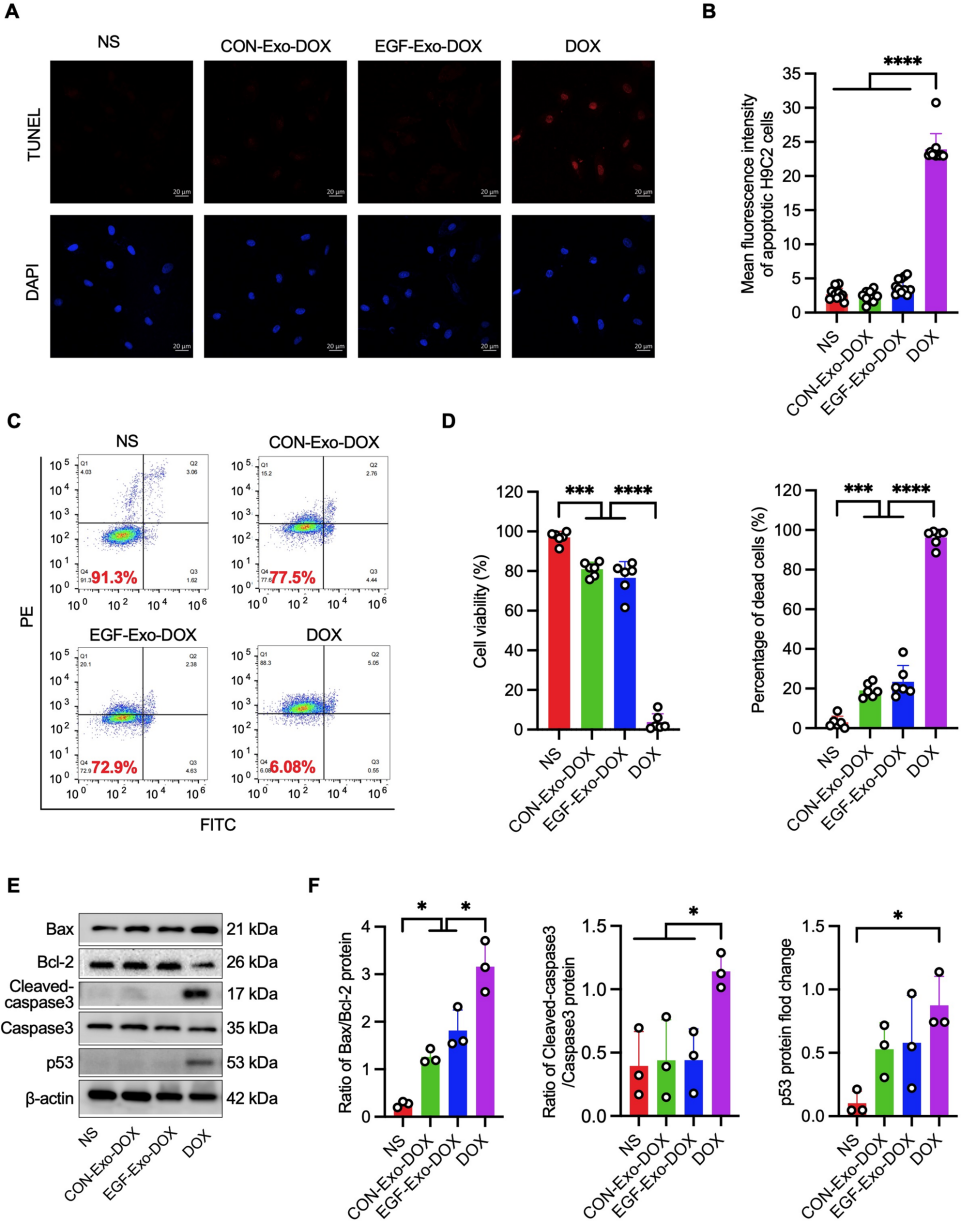
EGF-Exo-DOX mitigates DOX-induced cardiomyocyte apoptosis. H9C2 cells were incubated respectively with NS, CON-Exo-DOX, EGF-Exo-DOX, or DOX for 8 h, and apoptosis was analyzed. (**A**) Representative fluorescence images of H9C2 cells with TUNEL-positive cells (red), with DAPI-stained nuclei (blue). Scale bar = 20 μm. (**B**) Quantitative analysis of the MFI in (**A**) (*n* = 11). (C) Representative flow cytometry plots of H9C2 cells after each treatment (*n* = 6). (**D**) Quantitation of cell viability and the percentage of dead cells in (**C**) (*n* = 3). (**E**) The expression of Bax, Bcl-2, cleaved Caspase3, Caspase3, and p53, by Western blot. (**F**) Quantitation of the expression of apoptosis-related proteins p53, and the ratio of Bax/Bcl-2 and cleaved Caspase3/Caspase3 in (**E**). The expression of all target proteins was normalized to that of β-actin. All experiments were repeated 3 times in duplicates. Data are expressed as " Mean ± SD “, with * *P* < 0.05, ** *P* < 0.01, *** *P* < 0.001, and **** *P* < 0.0001

We have shown that EGF-Exo-DOX enhanced tumor suppressive potency and ameliorated cardiac dysfunction and adverse cardiac remodeling. To further evaluate the cardiotoxicity of EGF-Exo-DOX at the cellular level, A549 cells (1 × 10⁴) were seeded in 96-well plates and treated with free DOX at final concentrations of 1, 1.5, 2, 3, 5, and 10µM, or with EGF-Exo-DOX (1 × 10⁷ particles in 100µL, containing 0.07 µg DOX, equivalent to 1.29µM), for 8 h. MTT analysis showed a dose-dependent reduction in cell viability with free DOX. Notably, the percentage of apoptotic cells at 9.4µM free DOX was comparable to that observed with EGF-Exo-DOX treatment (Figure S6). Based on this similarity in cytotoxic effect, 9.4µM free DOX was selected as the equivalent dosage to evaluate the cardiac cytotoxicity of EGF-Exo-DOX. H9C2 cells (1 × 10⁶), which express lower levels of EGFR compared to A549 cells (Figure S7), were treated with 200µL normal saline (NS), CON-Exo-DOX, EGF-Exo-DOX (each containing 2 × 10⁸ particles, equivalent to 1.40 µg DOX, or 1.29µM), or 9.4µM free DOX for 8 h. TUNEL analysis showed a significantly higher percentage of apoptotic cells in the 9.4µM free DOX treatment group compared to the EGF-Exo-DOX, CON-Exo-DOX, and NS groups, which exhibited comparable baseline apoptosis levels (Fig. 6A and B). Flow cytometry analysis revealed a similar trend in cell viability, with live cells at 6.08% for free DOX, 72.9% for EGF-Exo-DOX, 77.5% for CON-Exo-DOX, and 91.3% for NS (Fig. 6C and D). In agreement, EGF-Exo-DOX treatment revealed significantly lower levels of pro-apoptotic proteins, including the ratios of Bax to Bcl-2 and cleaved-Caspase 3 to Caspase 3, and p53 compared to free DOX (Fig. 6E and F). Taken together, these results indicate that EGF-Exo-DOX reduces DOX uptake by cardiomyocytes, thereby mitigating DOX-induced apoptosis and overall cardiotoxicity.

### EGF-Exo-DOX demonstrates greater cellular uptake and apoptosis induction compared to Lipo-Dox

**Fig. 7:**
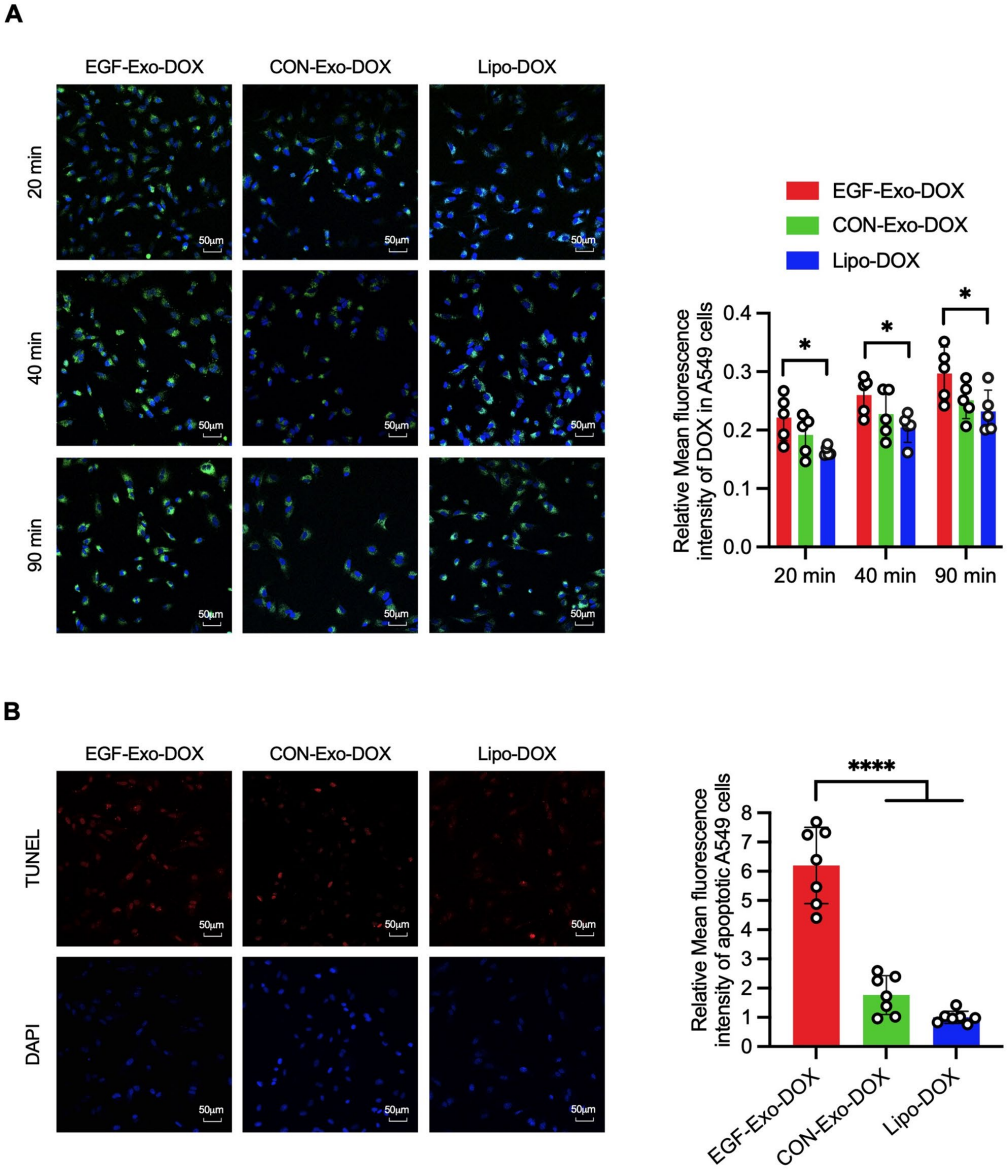
EGF-Exo-DOX enhances DOX delivery and induces higher apoptosis in A549 cells compared to Lipo-DOX. (**A**) Representative confocal fluorescence images showing DOX internalization (green) in A549 cells at 20, 40, and 90 min after treatment with EGF-Exo-DOX or CON-Exo-DOX or Lipo-DOX to A549 cells (left panel), with quantitation of the MFI (right panel). Nuclei were counterstained with DAPI (blue). (**B**) Representative confocal fluorescence images showing apoptotic cells in A549 cells at 4 h after treatment with EGF-Exo-DOX or CON-Exo-DOX or Lipo-DOX to (left panel), with quantitation of the MFI (right panel). Nuclei were counterstained with DAPI (blue). The scale bar = 50 μm. Data are expressed as “Mean ± SD”, with *p < 0.05 and ****p < 0.0001

To evaluate the anti-tumor efficacy of EGF-Exo-DOX, A549 cells were treated with EGF-Exo-DOX, CON-Exo-DOX, or Lipo-DOX at equivalent DOX concentrations. DOX internalization was assessed by confocal microscopy at 20, 40, and 90 min, while apoptosis was evaluated by TUNEL assay at 4 h. As shown in Fig. 7A, the MFI was significantly higher in EGF-Exo-DOX-treated cells than in those treated with Lipo-DOX at all observed time points, indicating enhanced DOX uptake; in contrast, MFI values were similar between CON-Exo-DOX and Lipo-DOX groups. At 4 h, the MFI reflecting apoptotic cells was significantly greater in the EGF-Exo-DOX group compared to both CON-Exo-DOX and Lipo-DOX (Fig. 7B), while no significant difference was observed between the latter two.

**Fig. 8:**
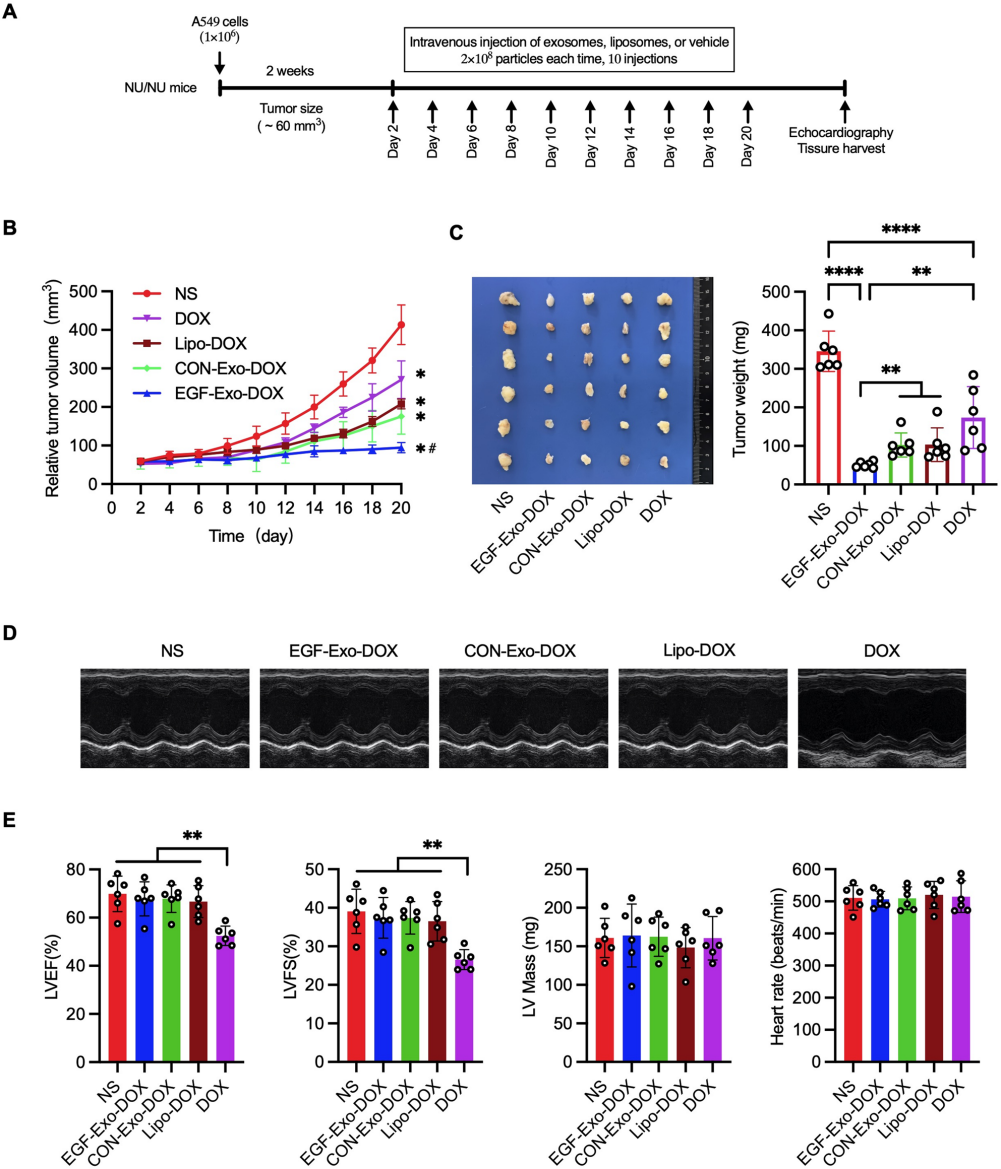
EGF-Exo-DOX exhibits greater anti-tumor potency than Lipo-DOX. A549 tumor xenograft mice were treated via tail vein injection every other day for a total of 10 doses of either NS, CON-Exo-DOX or EGF-Exo-DOX, lipo-DOX, or free DOX. (**A**) Schematic presentation of the therapeutic regimen. (**B**) Dynamic changes of tumor volume during the treatment (*n* = 6). (**C**) Gross images and quantitation of tumor weights after the treatment (*n* = 6). (**D**) Representative echocardiograms of tumor-bearing mice treated with NS, EGF-Exo-DOX, CON-Exo-DOX, lipo-DOX or DOX (*n* = 6). (**E**) Quantitation of LVEF, LVFS, LV Mass, and Heart rate in (**D**). Data are expressed as “Mean ± SD”, with * *P* < 0.05, ** *P* < 0.01, and **** *P* < 0.0001

To further evaluate the therapeutic efficacy of EGF-Exo-DOX, A549 tumor xenograft mice were treated via tail vein injection with EGF-Exo-DOX or CON-Exo-DOX (2⋅10^8^ particles in 200µL, containing 1.4 µg DOX), Lipo-DOX (200µL containing 1.4 µg DOX), or free DOX (1.4 µg) (Fig. 8A). While overall well-being and body weight remained comparable across all groups, tumor volumes and weights were significantly smaller in EGF-Exo-DOX group compared to the CON-Exo-DOX, Lipo-DOX, and free DOX. Tumor suppression was similar between CON-Exo-DOX and Lipo-DOX, both showing modest improvement over free DOX (Fig. 8B and C). LVEF and LVFS in the DOX group were significantly reduced compared to the NS, EGF-Exo-DOX, CON-Exo-DOX, and Lipo-DOX groups, which showed similar values among themselves. In contrast, LV mass and heart rate were comparable across all groups (Fig. 8D and E). These results demonstrate that EGF-Exo-DOX offers enhanced cellular uptake, cytotoxicity, and anti-tumor efficacy compared to Lipo-DOX.

## Discussion

DOX remains one of the most potent chemotherapeutic agents for tumor therapy, significantly improving the survival rate of cancer patients. However, many survivors are often burdened with, or succumbed to, the cumulative and dose-dependent cardiotoxicity [38, 39]. One of the most promising strategies to reduce cardiotoxicity is targeted delivery of exosomal encapsulated DOX. To leverage endogenous EGF-EGFR binding for efficient EGFR-mediated internalization and enhance targeting of tumors with diverse EGFR mutations, native EGF was displayed on the surface of exosomes derived from A549 tumor cells. These engineered exosomes demonstrated faster, EGFR-dependent cellular uptake of loaded DOX, resulting in a stronger apoptotic effect compared to Lipo-DOX in vitro. In vivo, EGF-Exo-DOX exhibited enhanced DOX accumulation in EGFR-positive xenograft tumors relative to the heart, demonstrating improved antitumor efficacy and reduced cardiotoxicity compared to Lipo-DOX, with no evidence of tumor metastasis or neoangiogenesis observed in major organs.

The therapeutic window of DOX refers to the range of plasma concentrations where it effectively kills cancer cells while minimizing damage to the heart [40, 41]. This window can be widened by directing the drug specifically to tumors and reducing its accumulation in other organs like the heart. Exosomes serve as an ideal carrier for achieving this drug distribution profile. The lower permeability of myocardial-endothelial monolayer, compared to other endothelial barriers, impedes exosomes from entering myocardium. This reduced myocardial accumulation can be amplified by the increased passive diffusion via the enhanced permeability and retention (EPR) effect in tumor vasculature, resulting in greater exosome build-up in tumors and less in the heart [24, 26, 29, 42–45]. This exosome distribution profile is supported by findings that systemically administered DOX-loaded exosomes exhibited increased accumulation in tumors with negligible accumulation in the heart, thereby highlighting the potential for enhancing the therapeutic index for tumors while reducing cardiotoxicity [24]. Our IVIS data support the limited accumulation of native exosome in the heart, with cellular uptake for CON-Exo being 25% lower in the heart compared to tumors. This favorable distribution profile, characterized by enhanced intratumor accumulation and reduced myocardial uptake, can be further improved through receptor-mediated cellular internalization, which is critical for DOX’s cytotoxic effects. By promoting cellular uptake, this mechanism minimizes exosome accumulation in the tumor stroma and enhances passive diffusion from the bloodstream into the tumor stroma [53]. This effect is evident in our EGFR-targeted delivery, where cellular internalization for EGF-Exo was 72% lower in the heart than in tumors (Fig. 2). Collectively, these effects contribute to improved anti-tumor effect and reduced cardiotoxicity.

Exosomes selectively enrich signature signaling molecules that can replicate the pathophysiological functions of their parental cells [43, 44]. Consequently, normal cell-derived exosomes (NDEs) are typically favored over tumor cell-derived exosomes (TDEs) for therapeutic applications to avoid the potential risk of tumorigenesis. However, TDEs have unique characteristics that make them effective drug delivery carriers for tumor therapy, and the therapeutic benefits they confer may counter or outweigh the potential risk of tumorigenesis [42, 46]. First, TDEs exhibit natural tropism toward their cell of origin or similar tumor microenvironments because of surface signature molecules retained from the parental tumor cells, enabling them to accumulate in tumor tissues and minimize off-target effects compared to synthetic liposomes. For instance, Qiao et al. reported that TDEs carry distinct patterns of surface integrins, which enable them to preferentially fuse with their parental cancer cells in vitro and home to original tumors in vivo. This resulted in improved cellular up-take by parental tumor cells and enhanced tumor suppression efficacy in xenograft nude mice [46]. In another study, TDEs encapsulating DOX-loaded porous silicon nanoparticles (PSiNPs), regardless of the origin of the parental cancer cells, demonstrated enhanced tumor stromal accumulation, increased cellular uptake, and greater cytotoxicity not only against bulk cancer cells but also against the side population of cancer stem cell in subcutaneous, orthotopic, and metastatic tumor models [47]. Second, TDEs have been shown to carry tumor-associated antigens inherited from their parental cells, which could prime the immune system through dendritic cells to elicit potent CD8^+^ T-cell-dependent anti-tumor effects in both syngeneic and allogeneic mouse tumor models [48].

In addition to the natural tropism of TDEs to tumors, exosome surfaces can be functionalized with EGFR homing peptides/EGF to further enhance targeted drug delivery to tumors [18, 49]. Ohno, et al. previously demonstrated that exosomes displaying GE11 or EGF on their surface, achieved through forced expression of fusion proteins in parental cells, underwent EGFR expression level-dependent internalization. Notably, both types of exosomes efficiently targeted EGFR-positive cells, only EGF-exosomes promoted cell proliferation in vitro, suggesting activation of EGFR signaling [50]. Consistent with these findings, our study also showed that EGF-Exo enhanced the proliferation of A549 cells in vitro, as indicated by increased cell viability (Figure S2B). Despite the mitogenic effect observed in vitro, EGFR remains one of the most widely used receptors for targeted drug delivery, including strategies such as Indium-tagged EGF, EGFR antibody-conjugated nanomicelles (PLGA-PEG/DOX@anti-EGFR), and EGFR homing peptides like GE11 and P22 [50–55]. These EGFR targeted delivery systems demonstrated rapid cellular uptake in an EGFR expression-dependent manner and increased tumor accumulation, thereby enhancing anti-tumor efficacy while reducing off-target toxicity. Similarly, EGF-Exo-DOX targeted tumor cell lines based on EGFR expression levels. Following systemic administration, it enhanced DOX delivery to xenograft A549 tumors while reducing cardiac accumulation, resulting in increased apoptosis rate within tumors and improved cardiac function, with no evidence of tumor metastasis and neoangiogenesis in major organs (Figs. 2, 3 and 4, S4, S5). That being said, the mitogenic effect associated with EGF-induced EGFR activation, observed in both previous studies and our own in vitro experiments, were not thoroughly evaluated in vivo and therefore remains valid concerns [56–58]. The absence of tumorigenic effects may be attributed to: (1) the limited number of exosome doses administered [49, 59, 60], (2) tumor type- and malignancy-specific responses, (3) enhanced cargo accumulation in tumors, which may have counteracted or outweighed the tumorigenic potential of EGFR activation, and (4) the relatively short observation period.

EGFR is targeted by two FDA-approved drug classes: tyrosine kinase inhibitors (TKIs) and monoclonal antibodies (mAbs), which effectively inhibit tumor growth and improve survival in EGFR-positive tumors but are limited by resistance due to EGFR mutations (e.g., L858R, T790M) [61–63]. In contrast, EGFR-targeted exosomes (EGF-Exo-DOX) demonstrated targeting capability across a broad spectrum of EGFR-expressing tumor cell lines (Fig. 1B and C), which most likely include those with EGFR mutations resistant to TKIs/mAbs since EGF bind to the extracellular domain of EGFR. This versatility enables effective drug delivery to both wild-type and mutant EGFR-expressing tumors, enhancing tumor-specific accumulation while minimizing off-target effects.

Our EGF-Exo-DOX formulation leverages the unique strengths of exosomes (such as their natural origin, biocompatibility, nanoscale size, low immunogenicity, and capacity to traverse biological barriers), the advantages of tumor-derived exosomes (TDEs) for tumor therapy (such as their intrinsic ability to home to parental tumor cells and immunomodulatory effects driven by tumor antigens), and active EGFR targeting. These combined attributes position EGF-Exo-DOX as a highly effective drug delivery platform, outperforming liposomal formulations for EGFR-positive tumor therapy. Indeed, studies from others and ours showed that, relative to Liposomal Dox, exosomal DOX was rapidly internalized into A549 cells (Fig. 7A) and redistributed to cytoplasm and nuclei of recipient cells, leading to increased cell apoptosis (Fig. 7B). In vivo, exosomal DOX demonstrated greater tumor penetration and accumulation, along with reduced cardiac accumulation, resulting in a reduced tumor burden and an improved cardiotoxicity profile [9, 28].

## Conclusions

In this study, EGF was displayed on the surface of exosome-derived from A549 tumor cells. These engineered exosomes were rapidly internalized by cell lines in an EGFR expression-dependent manner, exhibited enhanced accumulation in xenograft tumors, and reduced heart accumulation. When loaded with DOX, they remarkably reduced tumor burden and mitigated DOX-induced cardiotoxicity compared to Lipo-DOX, non-targeted exosomes, and free DOX (Graphic Abstract). Our findings suggest that TDEs bearing EGF on their surface hold promise as a readily available therapeutic option for patients with EGFR-positive tumors. However, before advancing to clinical trials, several key issues require thorough assessment: (1) The EGFR-dependent targeting of engineered exosomes should be confirmed using a single cell line with varying EGFR expression levels to rule out potential confounding effects from intrinsic differences in exosome uptake among different cell lines; (2) Standardized protocols for exosome characterization, bulk production, and DOX loading need to be developed; (3) The pharmacodynamic profile of the formulation must be investigated; (4) Optimal dose, timing/frequency, and number of doses must be determined; and (5) The long-term tumorigenic risk associated with tumor cell-derived exosomes and the activation of EGFR signaling pathways by EGF binding must be thoroughly evaluated.

## Supplementary Information

Supplementary Material 1

Supplementary Material 2

Supplementary Material 3

Supplementary Material 4

Supplementary Material 5

Supplementary Material 6

Supplementary Material 7

Supplementary Material 8

## Data Availability

The datasets used and/or analyzed during the current study are available from the corresponding author upon reasonable request.
