# Supplementary figures and images for: Exosomes displaying native EGF enhance doxorubicin’s therapeutic efficacy and reduce cardiotoxicity
Source: J Nanobiotechnology. 2026 Jan 27;24:179. doi: 10.1186/s12951-025-04002-9 (PMC12918744; doi:10.1186/s12951-025-04002-9)

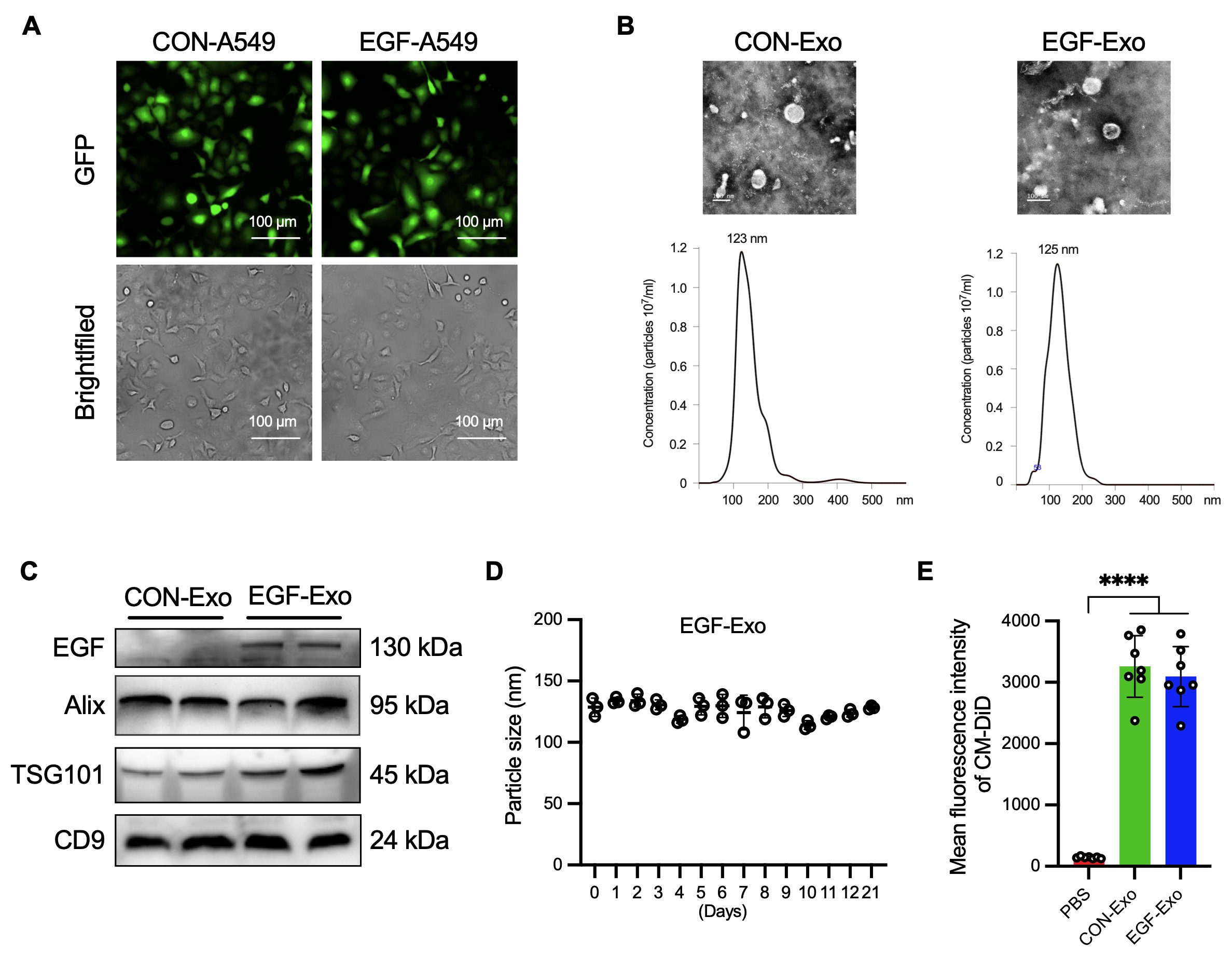

Supplement: Supplementary file 1 — Supplementary Material 1 [file 12951_2025_4002_MOESM1_ESM.jpg]

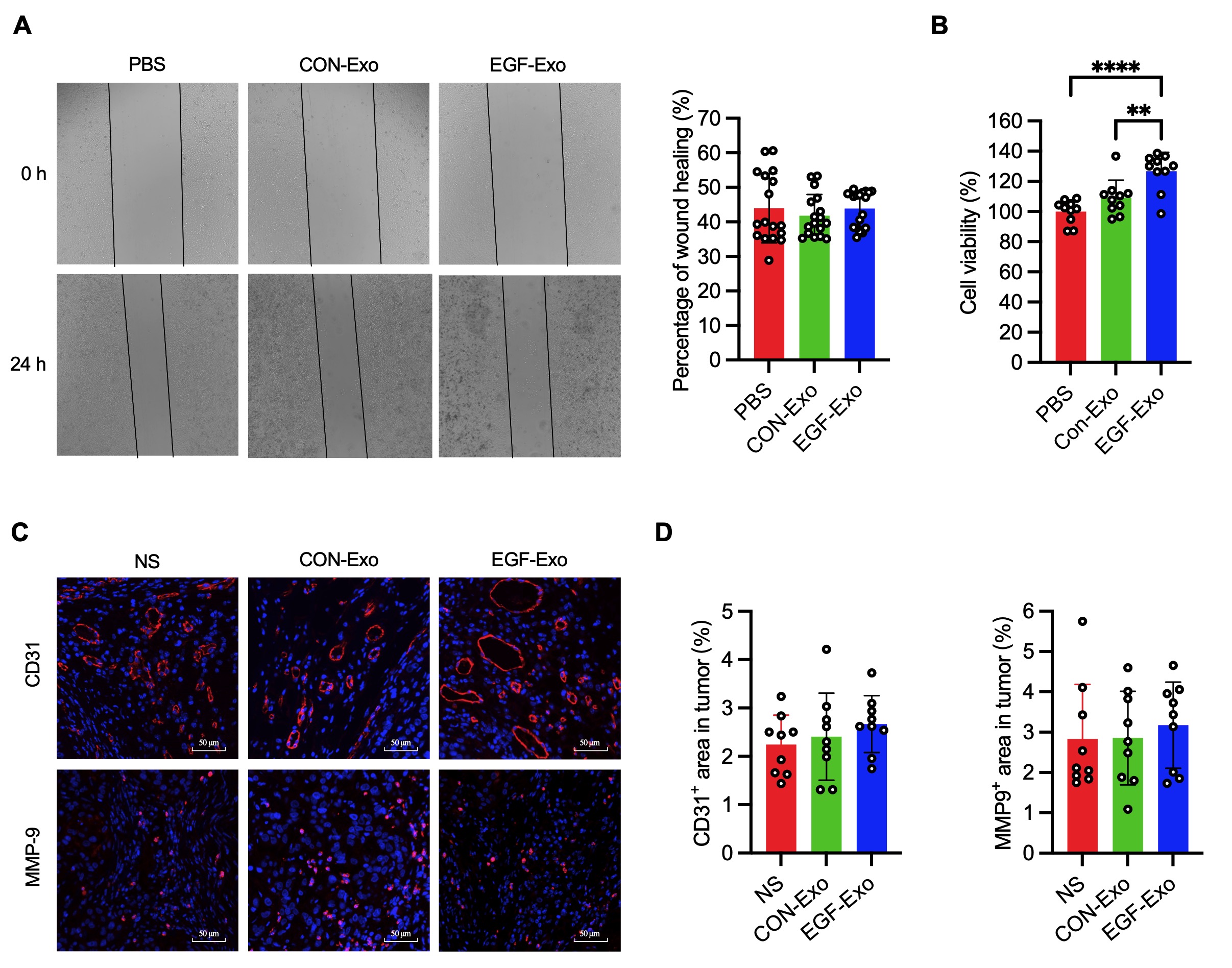

Supplement: Supplementary file 2 — Supplementary Material 2 [file 12951_2025_4002_MOESM2_ESM.jpg]

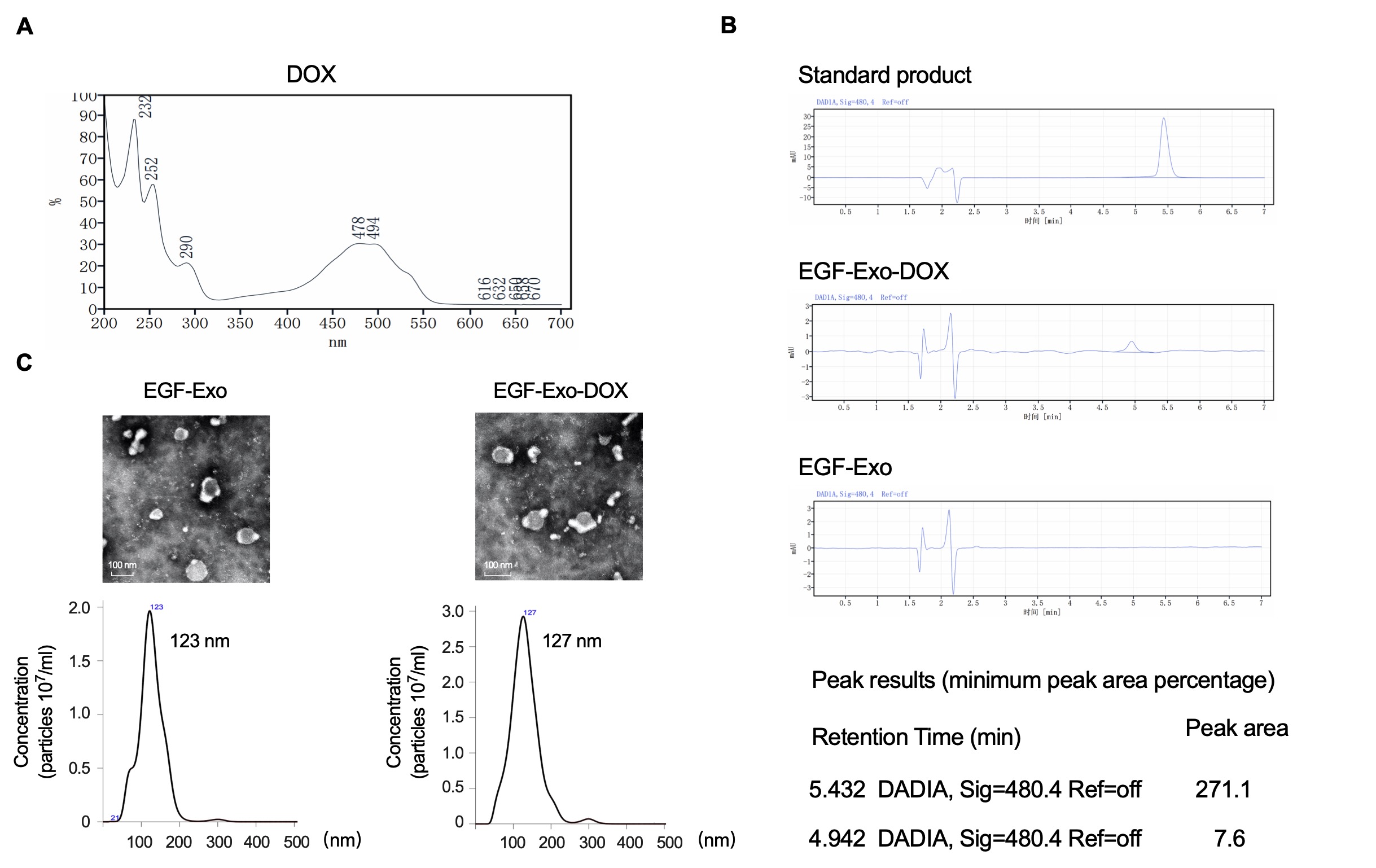

Supplement: Supplementary file 3 — Supplementary Material 3 [file 12951_2025_4002_MOESM3_ESM.jpg]

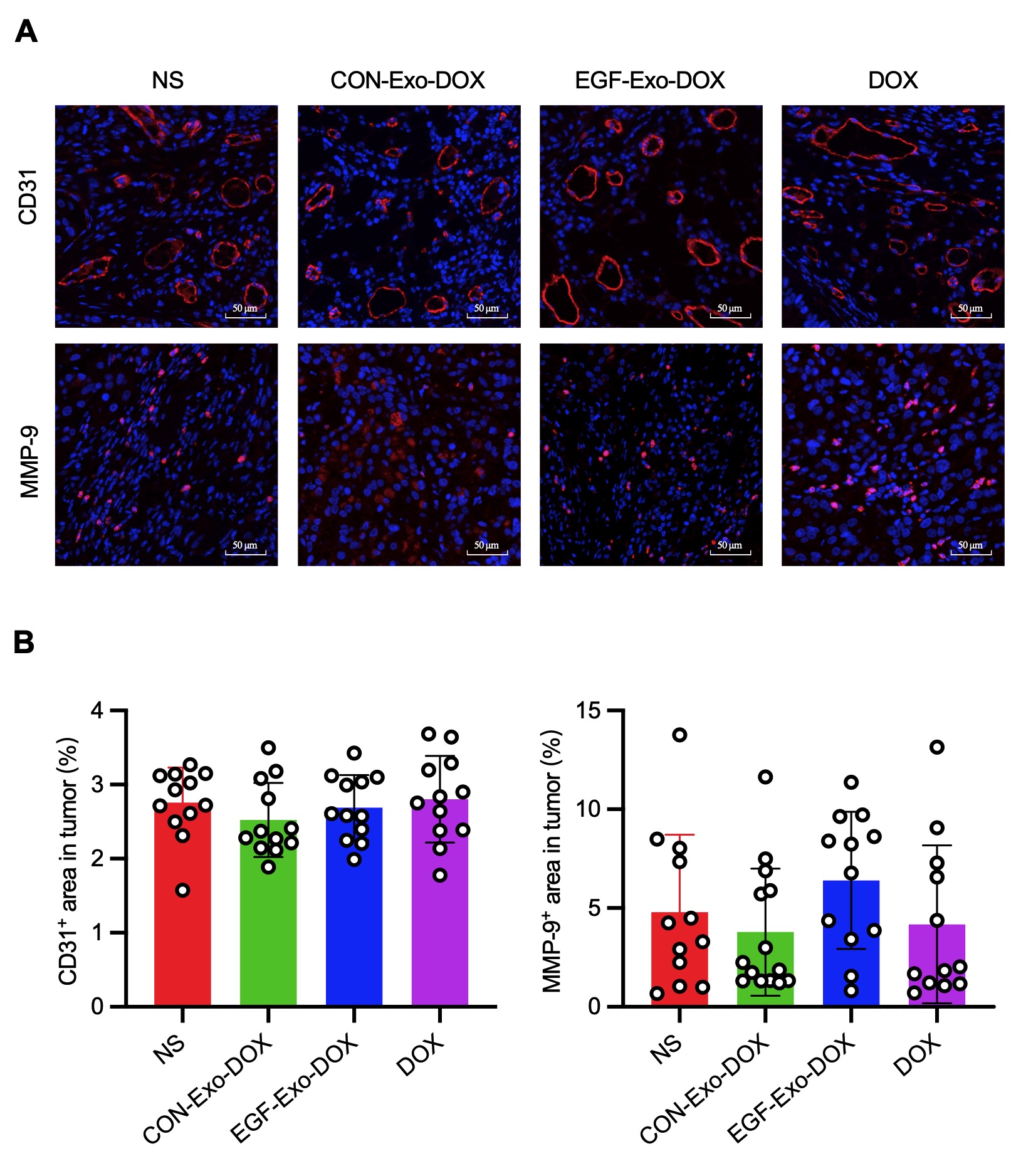

Supplement: Supplementary file 4 — Supplementary Material 4 [file 12951_2025_4002_MOESM4_ESM.jpg]

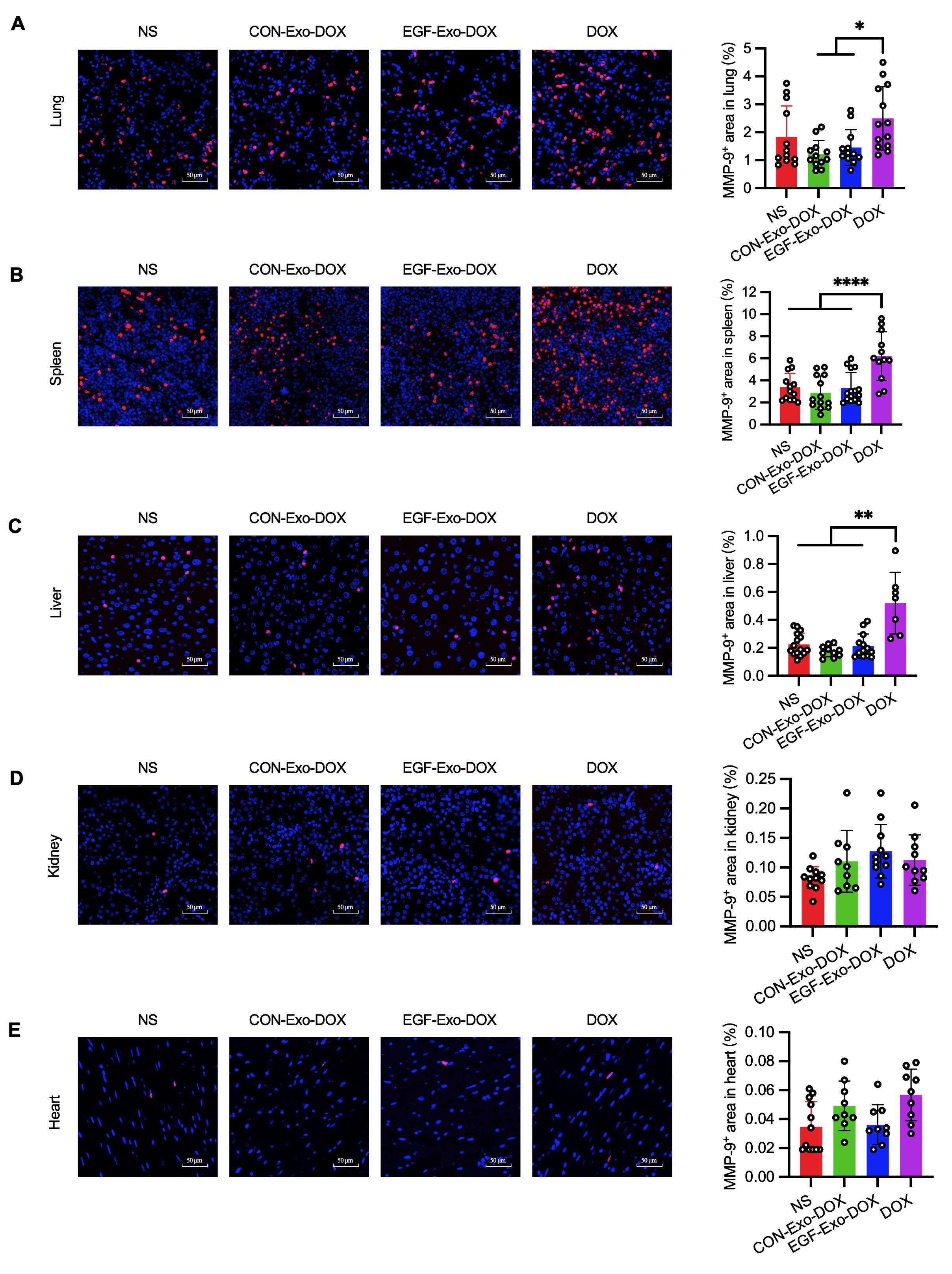

Supplement: Supplementary file 5 — Supplementary Material 5 [file 12951_2025_4002_MOESM5_ESM.jpg]

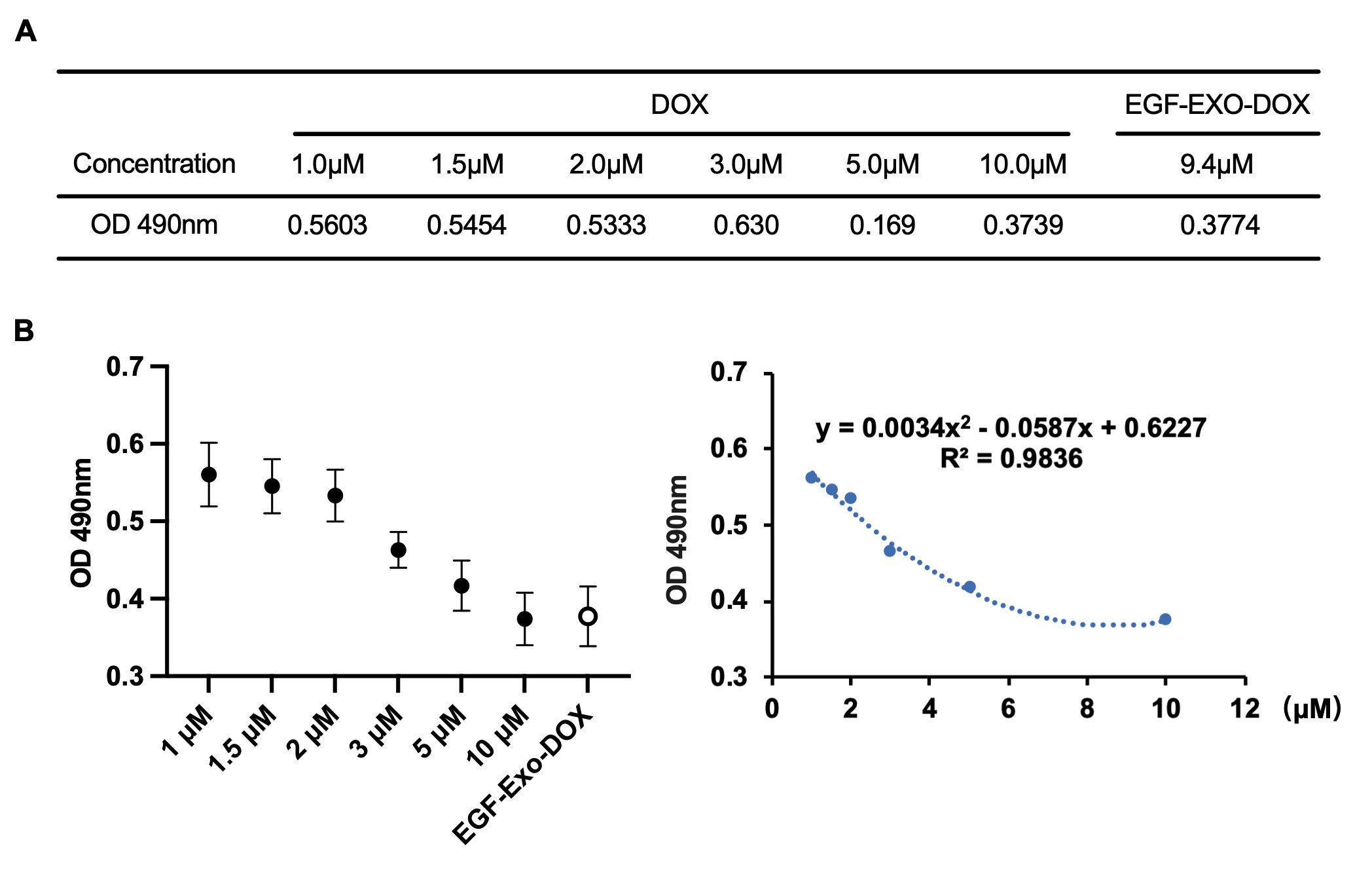

Supplement: Supplementary file 6 — Supplementary Material 6 [file 12951_2025_4002_MOESM6_ESM.jpg]

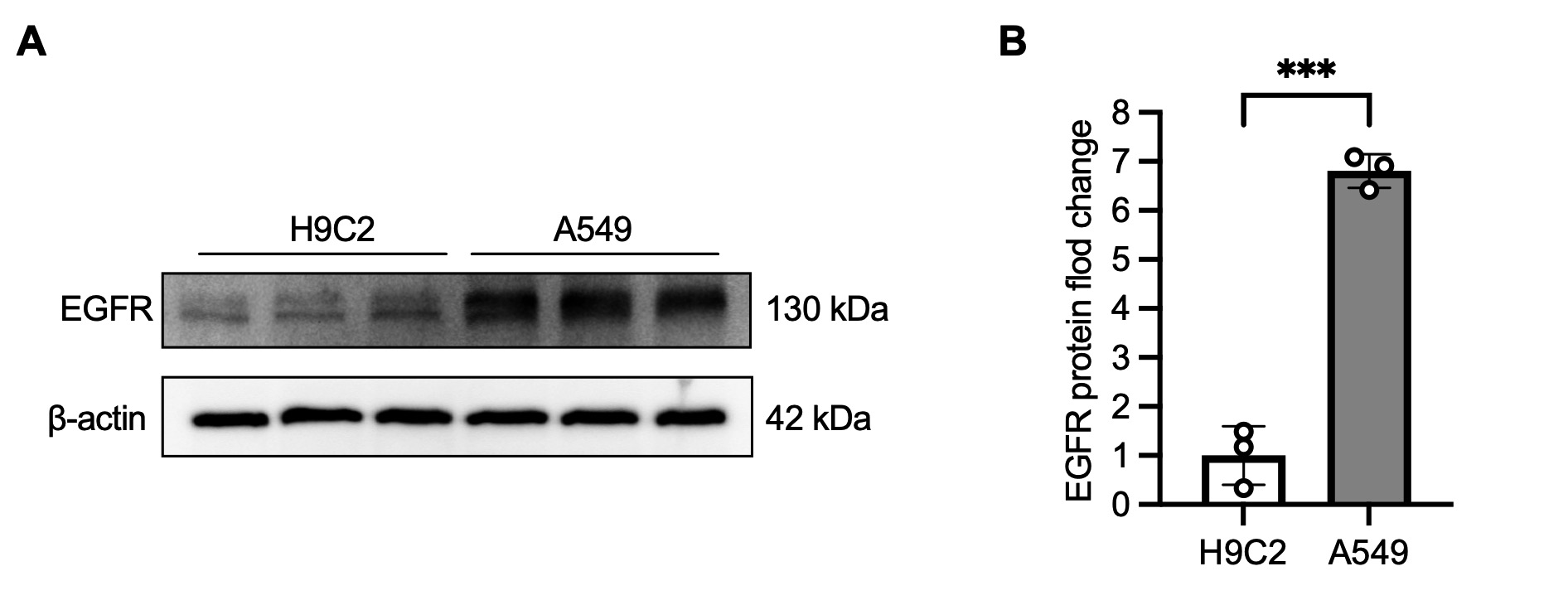

Supplement: Supplementary file 7 — Supplementary Material 7 [file 12951_2025_4002_MOESM7_ESM.jpg]
