# Supplementary material for: Exosomes displaying native EGF enhance doxorubicin’s therapeutic efficacy and reduce cardiotoxicity
Source: J Nanobiotechnology. 2026 Jan 27;24:179. doi: 10.1186/s12951-025-04002-9 (PMC12918744; doi:10.1186/s12951-025-04002-9)
Supplement: Supplementary file 8 — Supplementary Material 8 [file 12951_2025_4002_MOESM8_ESM.docx]

**Tables**

**Table S1. Primers for cloning plasmids displaying EGF_53_**

| Name | DNA sequence (5′-3′) |  |
| --- | --- | --- |
| LAMP Up | 5′-TATG**CTCGAG**TGCGGGGTCATGGTGTGCT-3′ | XhoI |
| LAMP Dn | 5′-ATCT**GGATCC**TTACAGAGTCTGATATCCAG-3′ | BamHI |
| EGF_53_ Up | 5′-TGTTCCACTTCAATGTTGAAGGCTTGCTCAGA  AAATGCCACTTGCCT-3′ |  |
| EGF_53_ Dn | 5′-GCAAGCCTTCAACATTGAAGTGGAACAATCT  GTCAAATTAAGTTCCA-3′ |  |

**Table S2. Primers used for qPCR gene expression analysis**

| Species | Gene symbol | Forward primer (5’-3’) | Reverse primer (5’-3’) |
| --- | --- | --- | --- |
| Human | Egfr | ACAGGCCACCTCGTCGG | CGTGAGCTTGTTACTCGTGC |
| Human | Gapdh | CATGAGAAGTATGACAACAGCCT | AGTCCTTCCACGATACCAAAGT |

**Table S3 Antibodies used in this study**

| Reagents | Manufacturer | Catalogue |
| --- | --- | --- |
| Anti-CD9 | Santa Cruz Biotechnology | SC-13118 |
| Anti-ALIX | Santa Cruz Biotechnology | SC-53540 |
| Anti-BCL2 | Santa Cruz Biotechnology | SC-7382 |
| Anti-TSG101 | Santa Cruz Biotechnology | SC-7964 |
| Beta-ACTIN | Santa Cruz Biotechnology | sc-47778 |
| Anti-BAX | Cell Signaling Technology | 2772 |
| Anti-CASPASE3 | Cell Signaling Technology | 9662 |
| Anti-cleaved CASPASE3 | Cell Signaling Technology | 9661 |
| Anti-p53 | Cell Signaling Technology | 9282S |
| Anti-EGF | Invitrogen | PA5-79185 |
| Anti-EGFR | Affinity biosciences | AF6043 |
